# Mutated ATP10B increases Parkinson’s disease risk by compromising lysosomal glucosylceramide export

**DOI:** 10.1007/s00401-020-02145-7

**Published:** 2020-03-14

**Authors:** Shaun Martin, Stefanie Smolders, Chris Van den Haute, Bavo Heeman, Sarah van Veen, David Crosiers, Igor Beletchi, Aline Verstraeten, Helena Gossye, Géraldine Gelders, Philippe Pals, Norin Nabil Hamouda, Sebastiaan Engelborghs, Jean-Jacques Martin, Jan Eggermont, Peter Paul De Deyn, Patrick Cras, Veerle Baekelandt, Peter Vangheluwe, Christine Van Broeckhoven, The BELNEU consortium, Chris van der Linden, Emke Maréchal, Patrick Santens, Wim Vandenberghe, Bruno Bergmans

**Affiliations:** 1 Laboratory of Cellular Transport Systems, Department of Cellular and Molecular Medicine, KU Leuven Campus Gasthuisberg, O&N I Herestraat 49-bus 802, 3000 Leuven, Belgium; 2 Center for Molecular Neurology, VIB, University of Antwerp, Universiteitsplein 1, 2610 Antwerpen, Belgium; 3 Institute Born-Bunge, Antwerp, Belgium; 4 University of Antwerp, Antwerp, Belgium; 5 Laboratory for Neurobiology and Gene Therapy, KU Leuven, Leuven, Belgium; 6 Leuven Viral Vector Core, KU Leuven, Leuven, Belgium; 7 Department of Neurology, Antwerp University Hospital, Edegem, Belgium; 8 Department of Neurology and Memory Clinic, Antwerp Hospital Network, General Hospitals Middelheim and Hoge Beuken, Antwerp, Belgium

**Keywords:** Parkinson’s disease, Massive parallel sequencing, ATP10B P-type ATPase, Endo-lysosomal lipid flippase, Loss-of-function, Glucosylceramide

## Abstract

Parkinson’s disease (PD) is a progressive neurodegenerative brain disease presenting with a variety of motor and non-motor symptoms, loss of midbrain dopaminergic neurons in the substantia nigra pars compacta and the occurrence of α-synuclein-positive Lewy bodies in surviving neurons. Here, we performed whole exome sequencing in 52 early-onset PD patients and identified 3 carriers of compound heterozygous mutations in the ATP10B P4-type ATPase gene. Genetic screening of a Belgian PD and dementia with Lewy bodies (DLB) cohort identified 4 additional compound heterozygous mutation carriers (6/617 PD patients, 0.97%; 1/226 DLB patients, 0.44%). We established that *ATP10B* encodes a late endo-lysosomal lipid flippase that translocates the lipids glucosylceramide (GluCer) and phosphatidylcholine (PC) towards the cytosolic membrane leaflet. The PD associated ATP10B mutants are catalytically inactive and fail to provide cellular protection against the environmental PD risk factors rotenone and manganese. In isolated cortical neurons, loss of ATP10B leads to general lysosomal dysfunction and cell death. Impaired lysosomal functionality and integrity is well known to be implicated in PD pathology and linked to multiple causal PD genes and genetic risk factors. Our results indicate that recessive loss of function mutations in *ATP10B* increase risk for PD by disturbed lysosomal export of GluCer and PC. Both ATP10B and glucocerebrosidase 1, encoded by the PD risk gene *GBA1*, reduce lysosomal GluCer levels, emerging lysosomal GluCer accumulation as a potential PD driver.

**Electronic supplementary material:**

The online version of this article (10.1007/s00401-020-02145-7) contains supplementary material, which is available to authorized users.

## Introduction

Parkinson’s disease (PD) affects over 10 million people worldwide [13, 88, 89]. Clinically, patients display a variety of motor (e.g. resting tremor, bradykinesia, rigidity, and postural instability) and non-motor symptoms (e.g. dementia, constipation, sleep disturbances), impeding their ability to perform basic everyday activities [22, 40]. Up to 30% of the patients with PD develop symptoms of dementia at a later stage of the disease [1]. To date, PD patients receive symptomatic treatment, though relief is temporary and not without adverse effects [32]. Neuropathological hallmarks of PD include the loss of midbrain dopaminergic neurons in the substantia nigra pars compacta and the presence of α-synuclein containing Lewy bodies in surviving neurons [19, 29, 108]. Although inherited PD is rare with an estimated frequency of 5–15%, the identification of high-penetrant mutations in causal genes in monogenic PD families have been instrumental in dissecting PD disease etiologies [61, 85, 114]. To date, four genes are responsible for autosomal recessive PD, associated with an early age at onset [58, 61, 107, 110]. Bi-allelic mutations in *PARK2* are the most common cause of autosomal recessive PD [86], explaining approximately 15% of early onset PD (EOPD) patients with an age at onset of 40–50 years [2], leaving a large fraction of EOPD patients genetically unexplained. In PD patient cohorts, genome-wide association studies (GWAS) identified over 70 loci with variable genetic contributions to PD risk [74]. Together these causal and risk genes represent only a small fraction of the genetic etiology of PD leaving both PD families and sporadic PD patients genetically unsolved. The knowledge acquired from the protein products of PD associated genes indicates that mitochondrial dysfunction, impaired intracellular trafficking, dysfunctional autophagy-lysosomal protein degradation pathways and α-synuclein aggregation are associated with neuronal death in PD [3, 17, 33, 44, 78, 117]. Several PD associated genes regulate membrane dynamics wherein mutations cause lipid homeostasis alterations associated with lysosomal dysfunction [34, 35, 65]. In particular, mutations in *GBA1* encoding the lysosomal enzyme glucocerebrosidase (GCase) increase the risk for PD with an estimated odds ratio of 5.4 [99]. GCase is involved in sphingolipid metabolism by catalyzing the breakdown of glucosylceramide (GluCer) and glucosylsphingosine (GluSph) to ceramide and sphingosine respectively in the lysosome [15, 30]. Up to 7–12% of PD patients carry mutations in *GBA1*, and hence *GBA1* is the most common genetic risk factor [23, 30, 100].

PD shows clinical, pathological and etiological overlap with dementia with Lewy bodies (DLB) and both diseases can be viewed as a continuum rather than dichotomous entities [46]. In DLB, the sequence of onset of dementia and parkinsonism is contrariwise compared to PD, with the initial cognitive deterioration resembling that of other dementias. DLB represents 10–25% of all dementias, only second to Alzheimer’s disease (AD) [47], and Lewy bodies are diffusely distributed throughout the cortices of the brain of DLB patients [105]. Novel candidate genes for either PD or DLB should be investigated in patients with the other phenotype as well, as the same genes may contribute to the development of both diseases, like *SNCA* and *GBA1* [45, 75, 81].

Via whole exome sequencing (WES) in 52 unrelated early-onset PD (EOPD) patients, we identified compound heterozygous mutations in the P4 transport ATPase class V type 10B gene (*ATP10B*) in 3 EOPD patients. The unaffected parents of these 3 EOPD patients each carried one variant in ATP10B, confirming *trans* configuration in the patients and as such recessive inheritance. Targeted resequencing of *ATP10B* in a Belgian PD and DLB cohort identified four additional patients carrying compound heterozygous mutations. We established that *ATP10B* encodes a late endo-lysosomal GluCer and phosphatidylcholine (PC) lipid flippase in which patient associated compound heterozygous mutations impair functional activities of ATP10B. Loss of ATP10B results in lysosomal dysfunction and sensitizes cortical neurons to cell death. Both ATP10B and GCase function in the same pathway regulating lysosomal GluCer content. Our genetics and functional data suggest that compound heterozygous *ATP10B* mutations contribute to risk for PD and DLB by a loss-of-function mechanism resulting in a dysregulated GluCer and PC homeostasis and lysosomal dysfunction.

## Materials and methods

### Belgian study populations

The PD cohort consisted of 617 unrelated patients (mean age at onset (AAO) 60.0 ± 11.5 years, 31.8% women, 18.6% with a positive familial history) and the DLB cohort consisted of 226 unrelated patients (mean AAO 70.8 ± 9.8 years; 32.7% female, 23.0% with a positive familial history) recruited in the framework of the Belgian Neurology (BELNEU) Consortium, a multicenter collaboration of neurology expertise centers in Belgium [20, 41]. Index patients were evaluated with a detailed clinical history of patients and family, clinical neurological examination, and neuroimaging. All PD patients fulfilled the National Institute of Neurological Disorders and Stroke (NINDS) diagnostic criteria for PD [40], whereas DLB patients were diagnosed in accordance with the established criteria for possible, probable or pathological DLB [71]. In the DLB cohort, 70 patients received a neuropathological diagnosis of DLB and 113 patients received a clinical diagnosis of probable DLB. Patients with a positive familial history have at least one first-degree relative presenting with PD or dementia. EOPD, was defined as PD with age at onset ≤ 50 years, and was observed in 120 patients of the PD cohort (mean AAO 43.4 ± 5.9 years, 24.2% women, 18.3% with a positive familial history). Of the EOPD patients, we collected detailed familial information and bio-sampled available family members. The complete PD cohort was genetically profiled for the 5 major PD genes (*SNCA*, *LRRK2*, *PARK2*, *PINK1* and *PARK7*) by means of Sanger sequencing for simple mutations, and multiplex amplicon quantification (MAQ, Agilent, Multiplicom, Niel, Belgium), quantitative real-time PCR or multiplex ligation-dependent probe amplification (MLPA) [50] for copy number variants [79]. Additionally, *VPS13C* was screened using a custom-designed gene panel (MASTR technology, Agilent, Multiplicom, Niel, Belgium) [42] followed by massive parallel sequencing on a MiSeq sequencing platform (Illumina, San Diego, CA, USA). We selected 52 unrelated EOPD patients (mean AAO 41.7 ± 6.7, 28.8% women, 23.1% with a positive familial history) with documented family history of disease and no disease-causing PD mutations for WES to search for novel PD genes.

A geographically-matched cohort, using self-reported ethnicity data, consisted of 598 unrelated control individuals with a mean age at inclusion (AAI) of 70.4 ± 7.9 years (67.7% female). Some control individuals were partners of the patients visiting a clinical neurology department, and who were screened for neurological or psychiatric antecedents and neurological complaints. The majority of the control individuals were community-dwelling volunteers, who tested with a score > 25 on a Montreal Cognitive Assessment (MoCA) [76] and without personal or familial history of neurodegenerative or psychiatric diseases.

### Whole exome sequencing

WES was performed on high molecular weight genomic DNAs of the selected 52 EOPD patients. The SeqCap^®^ EZ Human Exome Probes v3.0 (Roche) was used for exome enrichment for 23 patients followed by library sequencing on a NextSeq 500 platform (Illumina; ≥ 20 × coverage of RefSeq [80] coding region: 90.3 ± 1.88%). The SureSelect All Human Exon V5 + UTR enrichment (Agilent) was applied for 10 patients (≥ 20 × coverage of RefSeq [80] coding region: 87.3 ± 0.80%), the Nextera Rapid Capture Exome (Illumina) for 1 patient (≥ 20 × coverage of RefSeq [80] coding region: 61.18%) and the Nextera Rapid Capture Expanded Exome (Illumina), for 2 patient (≥ 20 × coverage of RefSeq [80] coding region: 78.13 ± 0.78%) followed by sequencing on a HiSeq 2000 platform (Illumina). The Burrows-Wheeler Aligner (BWA) [62] was used to perform the sequence alignment to the reference genome GRCh37 (hg19, UCSC Genome Browser). Variant calling was done with Genome Analysis Toolkit (GATK) Unified Genotyper [27, 72]. For 16 patients, exome enrichment was performed using the TargetSeq Exome Enrichment kit (Thermo Fisher Scientific, https://www.thermofisher.com) followed by sequencing on the SOLiD™ 5500xl Wildfire system (Thermo Fisher Scientific; ≥ 20 × coverage of RefSeq [80] coding region: 64.1 ± 6.75%). Here, read alignment and variant calling ware performed using the LifeScope™ Genomic Analysis Software (Thermo Fisher Scientific). GenomeComb, an in house developed package designed for analysis of massive parallel sequencing data, was used to annotate and select variants [91]. Because recessive PD genes are associated with an early onset age, we focused on homozygous and putative compound heterozygous variants (≥ two variants in one gene) with the following criteria: No occurrence or a minor allele frequency (MAF) ≤ 5% in Genome Aggregation Database (gnomAD) and < 25% in an in-house next generation sequencing (NGS) database of probands with distinct neurodegenerative brain disorders; variants with impact on protein level (non-synonymous missense, nonsense, frameshift, and splice site variants); high quality variants (coverage ≥ 15, genomic location outside repeat regions marked as simple repeats or micro satellites by RepeatMasker v3.0 [103]. Genes with autosomal recessive variants in 3–4 EOPD patients received the highest priority as well as genes with functional annotations related to known PD pathways.

### Whole genome sequencing

Whole genome sequencing (WGS) of EOPD patient DR621 and unaffected parents, subsequent read alignment to the human reference genome (NCBI build 37) and base and variant calling were performed by Complete Genomics™ Inc [31]. A minimum coverage of 20 × was obtained for 93.0 ± 2.2% of the bases. GenomeComb was used for variant annotation and selection [91]. Low coverage (< 20 ×) variants in addition to variants located in tandem repeats or high variability clusters were excluded from further analyses. We focused on exonic non-synonymous variants with a MAF less than 5% in the 1000 Genomes Project database or below 25% in an in- NGS database of probands with distinct neurodegenerative brain disorders. Variants were selected in line with the following inheritance patterns: X-linked, de novo, autosomal compound heterozygous recessive (in *trans*) or autosomal homozygous recessive. Validation of the selected variants in DNA samples of DR621 as well as variant genotyping in unrelated control individuals was performed using iPLEX Gold chemistry on the Sequenom MassARRAY platform followed by MALDI-TOF mass spectrometry, or by direct Sanger sequencing on an automated ABI3730xl DNA analyzer (Applied Biosystems). Primers were designed with either MassARRAY Assay Design software v.3.0.2.0 (Sequenom Inc) or Primer3. Genotypes were automatically called using MassARRAY Typer software v4.0 (Sequenom Inc) or the NovoSNP software package and visually checked according to GLP principles [116]. Variation combinations were assigned a lower priority for follow-up when present in aged control individuals. In case of X-linked variations, only genotypes of male individuals were accounted for. Homozygosity mapping of the WGS data using PLINK was performed as described elsewhere [55]. Homozygosity mapping did not reveal homozygous regions larger than 1 Mb in DR621, indicating that homozygous variants are unlikely to be pathogenic in the proband. Structural variants (SV) were called by Complete Genomics™ Inc [31] and a SV detection tool integrated in GenomeComb [91]. In silico disease-network analyses were performed using four algorithms: SUSPECTS, ToppGene, Endeavour and Biograph [4, 5, 63, 99]. These prediction programs rely on distinct combinations of features and metrics to link a predefined disorder with a set of candidate genes. The known genes for recessively inherited early-onset PD (*PARK2*, *PINK1*, and *PARK7*) and recessively inherited juvenile- or early-onset atypical parkinsonian syndromes (*FBXO7*, *ATP13A2* and *PLA2G6*) were used as training parameters. Candidate gene prioritization programs nominated *ATP10B* as the most likely disease gene in this family. Sanger sequencing was used to sequence the coding region and splice site junctions of all eight candidate genes in 120 EOPD patients.

### Targeted resequencing of *ATP10B*

PCR amplification of all 26 exons and flanking splice sites of *ATP10B* (NM_025153) was performed by a custom-designed amplicon-target PCR amplification assay (MASTR technology, Agilent, Multiplicom, Niel, Belgium) [42]. Amplicons were uniquely tagged; based on the Nextera XT shotgun library preparation protocol (Illumina, San Diego, CA, USA), containing sample-specific indices [59]. Libraries (*n* = 975) were pooled and sequenced in one run on the MiSeq platform using the MiSeq V3 chemistry, generating paired-end sequence reads of 300 nucleotides (Illumina, San Diego, CA, USA). After sample de-multiplexing, adapter clipping was performed with fastq-mcf [10] and sequence reads were mapped using the Burrows–Wheeler Aligner (BWA) [62] to a mini-genome, combining the target sequences extracted from the human genome reference sequence hg19. Primer sequences were clipped out using the sam_clipamplicons tool in Genomecomb [91]. The mean percentage > 20 × coverage of all target amplicons in 1441 samples (PD, DLB and control cohort) was 99.32 ± 1.92%. Sequence variants were called with GATKv2.4 UnifiedGenotyper and GATKv3.5 HaplotypeCaller [27, 72], and annotated using GenomeComb [91]. The option -dcov 1000 in GATK was used to downsample reads as of > 1000 reads per sample. We focused on homozygotes and compound heterozygotes (e.g. ≥ 2 variants per individual) with a frequency < 0.01%. Coding variants were numbered according to the GenBank Accession Number NM_025153 and amino acid changes according to the GenPept Accession Number NP_079429.

### Variant genotyping

Candidate variants identified in the WES/WGS data were validated and genotyped in relatives whenever possible by PCR amplification of genomic DNA followed by Sanger sequencing using the BigDye^®^ Terminator Cycle Sequencing kit v3.1 (Applied Biosystems) on an ABI3730 automated sequencer (Applied Biosystems). Primers were designed using the online Primer3 software [95]. Sanger sequencing was also used for the validation of rare (MAF < 5%) non-synonymous coding and splice site variants identified by targeted resequencing of *ATP10B*.

### Haplotype sharing analysis

Haplotype sharing between the relatives was analyzed by genotyping 15 polymorphic short tandem repeat (STR) markers surrounding *ATP10B* at chromosome 5q34: GATA139B09, D5S2090, D5S2013, D5S673, D5S2007, D5S1507, D5S2049, D5S412, D5S2038, chr5:159987161–159987527, chr5:160120387–160120617, D5S529, D5S422, D5S2066 and D5S2040. The STR markers were PCR amplified using fluorescently-labeled primers and size-separated using GeneScan 600 LIZ Dye Size Standard (Applied Biosystems) on an ABI3730xl DNA Analyzer (Applied Biosystems). Fragment lengths were scored using the in-house developed Local Genotype Viewer genotyping software (https://www.neuromicssupportfacility.be/).

### In silico splicing prediction

The splicing effect was evaluated in silico according to 4 splicing prediction programs (SpliceSiteFinder-like, MaxEntScan, NNSPLICE and GeneSplicer) integrated in Alamut Visual version 2.11.0 (Interactive Biosoftware, Rouen, France).

### Quantitative real time PCR

Total RNA of five substantia nigra and six medulla oblongata samples from six idiopathic PD patients without ATP10B mutations and four substantia nigra and medulla oblongata samples from four age- and gender-matched control individuals without neurologic pathology (provided by the Antwerp Biobank at the institute Born-Bunge) was extracted using the Ribopure kit (Ambion) and DNAse treated (Turbo DNAse kit, Ambion). Total RNA originating from a variety of human tissues (Life Technologies, AM6000) and brain regions, including substantia nigra (Clontech), cerebellum (Agilent Technologies), frontal cortex (Agilent Technologies & Stratagene), temporal cortex (Clontech & Biochain), parietal cortex (Aligent Technologies & Ambion), occipital cortex (Clontech), medulla oblongata (Biochain), striatum (Agilent Technologies), hippocampus (Clontech & Biochain), and basal ganglia were purchased. RNA concentrations and integrity were evaluated on a 2100 Bioanalyzer (Agilent Technologies). cDNA synthesis (Superscript III First-Strand synthesis, Thermofisher) was performed with both random hexamer and oligo (dT) primers. ATP10B expression was examined by SYBR green-based quantitative real time PCR (qRT-PCR, ATP10B ex10 FW: TCATCCTCATGTGCCTTATTGG & Rev: TGTTCTTCAAA GGTCCCATTCC; ATP10B ex17 FW: TCATGGAAACTGCACAGCATCT & Rev: CTGCAGCCGGTCTTCGAT). At least two reference genes were included in the experimental setup, based on their stability as calculated by Qbase+ (Biogazelle, HPRT1 FW: TGACACTGGCAAAACAATGCA & Rev: GGTCCTTTTCACCAGCAAGCT; GAPDH FW: ACGGGAAGCTTGTCATCAATG & Rev: GCATCGCCCCACTTGATTT; YWHAZ FW: CACAAGCAGAG AGCAAAGTCTTCTAT & Rev: AGCTTCTTGGTATGCTTGTTGTGA). All samples were run in triplicates. Normalization of ATP10B values and calculation of the relative mRNA expression levels was performed using Qbase+ software. Knockdown was verified with quantitative RT-PCR on RNA isolated from transduced and selected cells with the following primers: FW CTTCTACATGTTCCTCACAATGATCA, Rev GCTCAATGGAGACATACAAAGAGATG (human); FW CTT CTATATGTTCCTCACAATGATCA, Rev GCTCAATGGACACATACAAGGAGATC (mouse).

### Tissue culture maintenance

HeLa, Hek293T (Dharmacon, HCL4517), Im95m (JCRB cell bank, JCRB1075.1) and WM115 cell lines (Sigma, 91061232) were cultured in Dulbecco's Modified Eagle Medium (DMEM) culture media containing 1% l-glutamine and penicillin/streptomycin (Sigma-Aldrich; G7513 and P0781, respectively) as well as 10% fetal bovine serum (FBS; Life Technologies, 10270106). Im95m cells were cultured with 10 µg/ml insulin in the medium (Sigma-Aldrich; I9278). Cells were cultured for a maximum of 20 passages.

### Viral transductions

For viral transductions, 100,000 of either ATP10B negative HeLa, or endogenously expressing WM-115 or Im95m cells or 200,000 isolated cortical neurons were plated per well in 24-well plates. For overexpression, HeLa cells were transduced with lentiviral vectors coding for Cell Cycle Control Protein 50A (CDC50A) and ATP10B (encoding for the wild type (WT), catalytic dead mutants p.E210A and p.D433N, disease mutants p.R153*, p.G671R/p.N865K, p.V748L, p.E993A, p.I1038T, p.T161N, p.G393W, p.G6487R, p.I1222T and the polymorphism p.C217R or an enhanced green fluorescent protein (eGFP)-tagged WT and p.D433N variant). After lentiviral transduction, cells were selected with hygromycin (CDC50A, 200 µg/ml; Invitrogen, Ant-hg-1) or puromycin (ATP10B variants, 2 μg/mL; Invitrogen, Ant-pr-5) before confirmation by immunoblotting. For double transduction cells were first transduced with CDC50A vector, selected with hygromycin, and then transduced with the different ATP10B viral vectors. For knockdown, microRNA (mir) based short-hairpin lentiviral vectors were generated as described [82]. Viral vectors against 5 (human) to 7 (mouse) different target sequences were produced and validated for functionality. The most potent mir against each target were further used in this study (human mir2: ATGATTCAAGCTGCTGATATTG, human mir3: ACTTTGCCATCACCCGCTTTAA, human mir5: ACCTTAAGCTAGTACCTATATA, mouse mir5: TCCTGGTGATTCTGAACTGGAT, mouse mir7: CCCTAAGACAGTGCCTATACAT). A mir against firefly luciferase (mirFluc, ACGCTGAGTACTTCGAAATGTC) was used as control [82]. Transduced knockdown cell lines were selected with blasticidin (10 µg/ml, Invivogen).

### Transient transfection

For the subcellular localization of ATP10B, stable eGFP-ATP10B WT or eGFP-ATP10B p.D433N cell lines were transiently transfected with either an endo-/lysosomal marker (Rab5-mCherry, Rab7-mCherry, LAMP1-mCherry) or an endoplasmic reticulum (ER) marker (Serca-mCherry) using Lipofectamine 2000 (Thermo Fischer Scientific, 11668-027, at a ratio of 3:1) for 8 h in FBS free media prior to overnight incubation in full culture media. The cells were washed with PBS (Life Technologies, 14190169) and fixed (30 min, 37 °C) with 4% paraformaldehyde (PFA, Affymetrix, 199431LT).

### Neuron Isolation and experimental culture

Primary cortical neurons were derived from E16 FVB/N mice embryos. Pregnant mice were sacrificed on gestation day 16 by cervical dislocation. E16 mice pup brains were collected and placed in a dish containing calcium- and magnesium-free Hanks’ Balanced Salt Solution (HBSS, Life Technologies, 14180-046) on ice. Both cerebral hemispheres were separated from the cerebellum. Meninges were removed from the cerebral hemispheres and brain cortices were dissected. Brain cortices were collected, washed twice and digested with 0.05% trypsin (Life Technologies, 25300-054, 10 min at 37 °C). Trypsin reaction was stopped by adding 7 ml of HBSS and 1 ml of horse serum. Cells were separated by pipetting and filtration through a 40 μm cell strainer (Falcon, 352340). Cells were centrifuged for 5 min at 1000 rpm (4 °C), the supernatant removed and the pellet suspended in 5 ml Dulbecco’s Modified Eagle Medium (DMEM; Sigma-Aldrich, D6546) + GlutaMAX (Life Technologies, 31966-021) containing 5% horse serum (Life Technologies, 26050-088) and 20 mM glucose (Sigma-Aldrich, 8270). Primary cortical neurons were plated in the relevant well plates, coated with poly-d-lysine (Sigma-Aldrich, P6407). After an overnight incubation, cell medium was exchanged for Neurobasal medium (Life Technologies, 21103-049) supplemented with 2% B27 (Life Technologies, 17504-044) and 2 mM l-glutamine (Life Technologies, 25030-24). At 3 days in vitro (DIV) the neurons were transduced with two independent ATP10B targeting mir’s, utilizing mirFluc as a control. For recovery of ATP10B expression within experimental knockdown conditions, lentiviral based overexpression of ATP10B or p.D433N was performed on DIV 4. Fluc was used as a control. At DIV 6, neurons were treated and experiments were terminated at DIV 7.

### Treatments and reagents

Cell lines and isolated neurons were treated with indicated doses of rotenone (dimethyl sulfoxide (DMSO), 0–10 µM; Sigma-Aldrich, R8875), zinc (ZnCl_2_, 0–10 µM, Sigma-Aldrich, Z0152), the proteasome inhibitor bortezomib (phosphate buffered saline (PBS), Bort, 100 nM; Bio-Connect BV, 354938) or manganese (PBS, MnCl_2_ 0–1 mM; CASP, 25,605) for 48 h. Apoptosis was blocked by a 1 h pre-incubation with the caspase inhibitor Zvad-fmk (DMSO, Zvad, 50 µM; Bachem, N1560-0005). Lysosomal functionality was blocked by a 1 h pre-treatment with bafilomycin (BAF A1, 50 nM or 1 µM; Sigma-Aldrich, B1793-10UG). In non-treated conditions, vehicle was used as a control. For the experimental use of lipids, lipid stocks were prepared in 100% chloroform and a lipid film was formed by evaporation under nitrogen. Finally, lipids were re-suspended in either 95% ethanol (translocation assays) or reaction buffer supplemented with 10 mM of 3-[(3-cholamidopropyl)dimethylammonio]-1-propanesulfonate hydrate (CHAPS, ATPase assay).

### Autophosphorylation assay

HeLa microsomes were prepared according to [49]. The assay was performed as previously described [48] with minor adaptations. Firstly, during microsomal preparation ATP10B was isolated in styrene maleic acid co-polymer (SMA) lipid particle-based microenvironment (SMALP), whereby the SMA co-polymer (PolySoMA, Uispac) was added to a final concentration of 2.5% (wt/vol) before the final ultracentrifugation step. Secondly, for the detection of ATP10B phospho-enzyme a reaction time of 5 min was used.

### ATPase assay

HeLa microsomes were prepared [49]. ATPase activity was assessed using the commercially available ADP-Glo MAX assay (Promega). Briefly, 5 µg of microsomes were plated per well in a white 96-well plate, in 50 mM 3-(*N*-Morpholino)propanesulfonic acid (MOPS), 100 mM KCl, 11 mM MgCl_2_, 1 mM Dithiothreitol (DTT), 195 µM *n*-dodecyl-d-maltoside (DDM, pH 7, KOH) and allowed to equilibrate on ice for 1 h, placed at 37 °C for 5 min prior to the stimulation of ATPase activity by the addition of 5 mM ATP for 30 min (37 °C). Reactions were terminated by Glo MAX assay reagent addition and luminescence was monitored after 1 h using a Flexstation 3.0 microplate reader. To determine the effect of lipid substrate addition on ATP10B ATPase activity, 10 µg of microsomes were plated per well in a white 96-well plate, in 50 mM 4-(2-hydroxyethyl)-1-piperazineethanesulfonic acid (HEPES), 150 mM KCl, 12.5 mM MgCl_2_, 1 mM DTT (pH 7, KOH) with 500 µM phosphatidylcholine, 500 µM glucosylceramide or the combination (250 µM of each lipid) and reactions were performed as described above.

### Lipid translocation assay

HeLa microsomes were prepared according to [49]. ATP-dependent nitrobenzoxadiazole (NBD) phospholipid translocase activity was measured as described by [77], with some minor adaptations. In brief, microsomal membranes, harvested from stable HeLa cells overexpressing ATP10B WT or p.D433N, were assayed for ATP10B-dependent flippase activity using a back-extraction method. Equal volumes of microsomes, NBD-lipids (10 µM) and ATP regenerating system (without ATP) were mixed together and incubated for 1 h on ice to allow incorporation of the NBD-lipid into the cytosolic membrane leaflet. Subsequently, samples were incubated at 37 °C and after 2 h, ATP was added to stimulate ATP10B-dependent flippase activity. At hourly time points, 3 aliquots of 15 μl each were removed and added to one tube (A) containing 7.5 μl of buffer H (10 mM HEPES, pH 7.5, 150 mM NaCl) and two tubes (B, C) containing 7.5 μl of 3% fatty acid-free bovine serum albumin (BSA, Merck-Millipore) in buffer H. After 5 min on ice, 177.5 μl of ice-cold buffer H was added to each tube, and samples A and B were centrifuged at 150,000×*g* for 15 min (4 °C), while sample C was left on ice. The supernatants from samples A and B and the entire sample C were transferred to a 96-well plate and mixed with 200 μl 2% Triton X-100 in buffer H. The NBD fluorescence of each sample was measured using a fluorescence plate reader (FlexStation, Molecular Devices) with excitation at 467 nm and emission at 534 nm (cut-off 530 nm). The percentage of NBD-phospholipid in the cytosolic membrane leaflet was calculated as described in [77]. To investigate the effect of disease mutations or substrate specificity experiments were performed as 4 h endpoint measurements. For substrate specificity, competition experiments were performed with the aforementioned procedure; however the membrane fraction were incubated with NBD-PC or NBD-GluCer alone or in combination with a spectrum on non-NBD labeled lipids (10 µM), equilibrated on ice for 1 h followed by 37 °C for 2 h. Flippase activity was activated by the addition of ATP and terminated 2 h later. Samples were collected and assessed as described above.

### Co-immunoprecipitation

HEK293T cells were lysed 48 h post-transfection in lysis buffer (25 mM Tris–HCl, pH 8.0, 150 mM NaCl, 2% Nonidet P-40, supplemented with complete protease (Roche) and Phospho-STOP (Sigma) inhibitor mixtures) for 30 min on ice and cleared by centrifugation (10 min, 20,000×*g*). Protein concentration of the supernatant was determined using a bicinchoninic acid (BCA) protein assay (Pierce™). 1 mg/ml of all cell lines was incubated overnight in the presence of protein G Dynabeads™ (Thermo Fisher Scientific) and HA antibody (Cell signaling, 3724) or normal rabbit IgG antibody (Santa Cruz, sc-2027) at 4 °C on a rotating device. Afterwards, the beads were collected and washed repeatedly with the lysis buffer. Finally, beads were re-suspended in lysis buffer supplemented with 4 × LDS sample buffer and 100 mM DTT, boiled for 10 min at 95 °C, and loaded on 4–12% Bis–Tris NuPAGE gels (Life Technologies).

### Split luciferase assay

A protein complementation assay based on firefly luciferase was used to show interaction between ATP10B and CDC50A, with eGFP as a control. Plasmids for the N- and C-terminal parts of luciferase were generated that fused to ATP10B WT, CDC50A and eGFP was used as a control. 20,000 HEK293T cells were plated 20,000 cells/well in a 96-well plate and transfected with different combinations of the aforementioned plasmids. Luciferase activity was measured 48 h after transfection using the ONE-Glo Luciferase Assay System (Promega) and is used as a measure for protein interaction between the N- and C-terminal parts of luciferase.

### Immunoblotting

HeLa microsomes were prepared according to [49] and immunoblotting was performed as previously described [113]. For total cell lysate investigation, cells were lysed using RIPA buffer (ThermoFisher Scientific, 89900) and DNA excluded by centrifugation (15,000×*g* for 15 min). Briefly, western blots of typically 20–40 µg of protein were ran on 4–12% Bis/Tris gel (NuPage, Thermo Scientific, NP0323BOX) and transferred to a 0.45 µm PVDF membrane (Immobilon-P, Thermo Scientific, 88518) and probed for ATP10B (Sigma-Aldrich, HPA034574), CDC50A (anti-FLAG antibody; Sigma-Aldrich, F3165). Cell death was assessed using a cleaved caspase 3 antibody (Cell signaling, 9661). All blots were probed for either GAPDH (Sigma-Aldrich, G8795) or β-actin (Sigma-Aldrich, C6198) as a loading control. Detection was performed using HRP-conjugated secondary antibodies (BIOKE, 7074S and 7076S). Detections were performed on a Bio-Rad Chemidoc Imager with Pierce ECL Western Blotting Substrate (Thermo Fisher Scientific, 32106). For co-immunoprecipitation experiments, PVDF membranes (Hybond P; Amersham Biosciences) were blocked in 5% skimmed milk in PBS and probed with primary antibodies against HA-tag (Covance, MMS-101P) and FLAG-tag (Novus Biologicals, NBP1-06712).

### Immunofluorescence

After fixation with 4% PFA, cells were washed twice with PBS containing 0.5% tween 20 (PBS/T; Sigma-Aldrich, P1379) and permeabilized with 0.1% Triton X-100 (Sigma-Aldrich, T9284) and 0.02% SDS (Acros Organics, 230425000) containing PBS/T for 20 min. To minimize non-specific binding, 0.1 M Glycine in PBS/T was added to the cells after washing followed by blocking in PBS/T containing 1% BSA and 2% FBS. After washing, cells were incubated overnight at 4 °C with various primary antibodies: Rab11 (Thermo Fisher Scientific, 71-5300), Rab5 (Santa Cruz Biotechnology, Sc-46692), EEA-1 (Becton Dickinson, 610457), CD63 (BIO-CONNECT Diagnostics, 11-343-C100), LAMP-1 (Abcam, ab24170), Rab7 (Abcam, ab137029 and ab25245), LAMP2 (Abcam, ab25339 and ab25631), ATP10B (Sigma-Aldrich, HPA034574) or cleaved caspase 3 (Cell Signaling, 9661) diluted according to manufacturer’s recommendations in PBS/T containing 0.1% BSA and 0.2% FBS. Finally, after the samples were washed with PBS-T, cells were incubated with Alexa Fluor dyes (Thermo Fisher Scientific, 1/2000) for 30 min. Nuclei were stained with DAPI (Sigma-Aldrich, D9542-10 mg). Cells were visualized using an LSM780 confocal microscope.

### Cell viability

Cells were seeded at 5000 cells per well in a 96-well plate. After treatment, cells were washed with PBS and incubated with 0.01 mg/ml MUH (4-methylumbelliferyl heptanoate; Sigma-Aldrich, M2514; dissolved in PBS) for 30 min at 37 °C. Fluorescence was measured with a Flexstation 3.0 plate reader (Molecular Devices, Wokingham, UK) at an excitation of 355 nm, emission of 460 nm, and cut off value of 455 nm.

### Cell death

HeLa cells treated with rotenone or MnCl_2_ were assessed for cell death induction as previously described [48]. Briefly, treated cells were collected at 48 h by trypsinization, PBS-washed and exposed to 1 µg/ml propidium iodide (PI; Sigma-Aldrich, P4170-25MG) for 5 min. PI positivity was captured using an Attune flow cytometer (Life Technologies). Neuronal cell apoptosis was assessed by cleaved caspase 3 staining in accordance with the manufacturer’s guidelines (Cell Signaling, 9661). Cortical neurons were plated with or without cover slips. Cells plated without coverslips were ultimately assessed by flow cytometry using an Attune Flow Cytometer. Samples seeded on coverslips were imaged using a LSM780 confocal microscope (Leica). DAPI staining was used to visualize the nucleus.

### TUNEL staining

Terminal deoxynucleotidyl transferase dUTP Nick-End Labeling (TUNEL) was assessed according to the manufacturer’s protocol for the Click-iT Plus TUNEL assay (ThermoFisher, C10617). Images were captured using a Leica LSM780 confocal microscope.

### Fluorescein isothiocyanate-dextran based lysosomal pH

WM115 cell lines were plated in 12 well plates (100,000 cells per well) and allowed to attach overnight 37 °C. Cells were then exposed to 50 µg/ml fluorescein isothiocyanate (FITC)-dextran for 72 h. Samples were then washed, placed in fresh media, and treated with the appropriate stressors (1 µM Rotenone and 1 µM BAF A1) for 4 h. Samples were then collected by centrifugation (450×*g*, 5 min) and washed in PBS containing 1% BSA. Cells were finally re-suspended in 50 µl of PBS/BSA and FITC dual emission assessed by flow cytometry (excitation 488 nm, emission 530 (BL1) and 600 (BL2) using an Attune NXT flow cytometer (invitrogen). The emission (BL1/BL2) of all samples were compared to the signals obtained for control cells re-suspended in monensin (100 µM) containing Britton Robinson buffer with increasing pHs (3.0–8.0).

### Lysosomal degradative capacity (DQ-BSA)

Cells were seeded in 12-well plates (1.0 × 10^5^ cells per well) and the next day, cells were pre-treated with rotenone for 1 h at 37 °C. 50 nM BAF A1 was used as an internal control. Subsequently, 5 µg/ml DQ Green BSA was added to the cells for a further 3 h (37 °C). Finally, cells were collected and the mean fluorescent intensities of 10,000 events were assessed using an Attune Nxt (Thermo Scientific) flow cytometer. For the assessment of lysosomal degradation capacity in isolated cortical neurons, cells were seeded in 12-well plates containing cover slips (3.0 × 10^5^ cells per well) and the next day ATP10B was knocked down via lentiviral transduction. mirFluc was used as an internal transduction control. 48 h post lenti-viral exposure. ATP10B WT, p.D433N or Fluc were over-expressed via lentiviral transduction and allowed to incubate for a further 24 h at 37 °C. Following rescue, cells were treated with 10 µg/ml DQ-BSA for 1 h prior to treatment with rotenone (50 nM), and incubated further for 23 h at 37 °C. Cells were subsequently fixed, DAPI stained and images captured via confocal microscopy (Zeiss LSM780).

### Lysosomal membrane integrity

To assess lysosomal membrane integrity, WM-115 cells were seeded in 12-well plates (1.0 × 10^5^ cells per well) and the next day, cells were incubated with 5 µg/ml acridine orange (dissolved in media) for 15 min at 37 °C. Thereafter, medium was discarded, cells were washed and fresh medium was added. Cells were then treated with rotenone or the positive control BAF A1 (1 µM) for 4 h at 37 °C. Finally, cells were collected and resuspended in PBS containing 1% BSA. The mean fluorescence of 10,000 events was captured using an Attune Nxt (Thermo Scientific) flow cytometer.

### Statistics

To investigate association between recessive *ATP10B* mutations and PD, mutation frequencies were statistically compared between the early-onset patient group and control individuals using Fisher’s exact statistics. *ATP10B* gene expression levels in PD patient brains versus brains of neurologically healthy individuals were statistically compared using a Mann–Whitney *U* test. Statistical significance of lipid translocation, cell death, and viability were performed by *t* test and one/two way analysis of variance within GraphPad Prism 6.01. Pearson’s coefficients were generated using Image J. Unless otherwise specified, data are represented as the average ± standard error of the mean (SEM) of a minimum of three independent experiments.

### Ethical assurances

The genetic studies were approved by the ethic committee of the Antwerp University Hospital and the University of Antwerp. Clinical protocols were approved by the ethics committees of the main participating hospitals i.e. the Hospital Network Antwerp and the University Hospital Antwerp, as well as by the ethical review boards of the participating general hospitals which are participating via the BELNEU consortium. All human biological samples were collected in accordance with the written informed consents signed by the participants. All mouse primary neuron experiments were carried out in accordance with the European Communities Council Directive of November 24, 1986 (86/609/EEC) and approved by the Bioethical Committee of the KU Leuven (Belgium) (ECD project P185-2014).

## Results

### Identification of compound heterozygous ATP10B mutations in patients

**Fig. 1:**
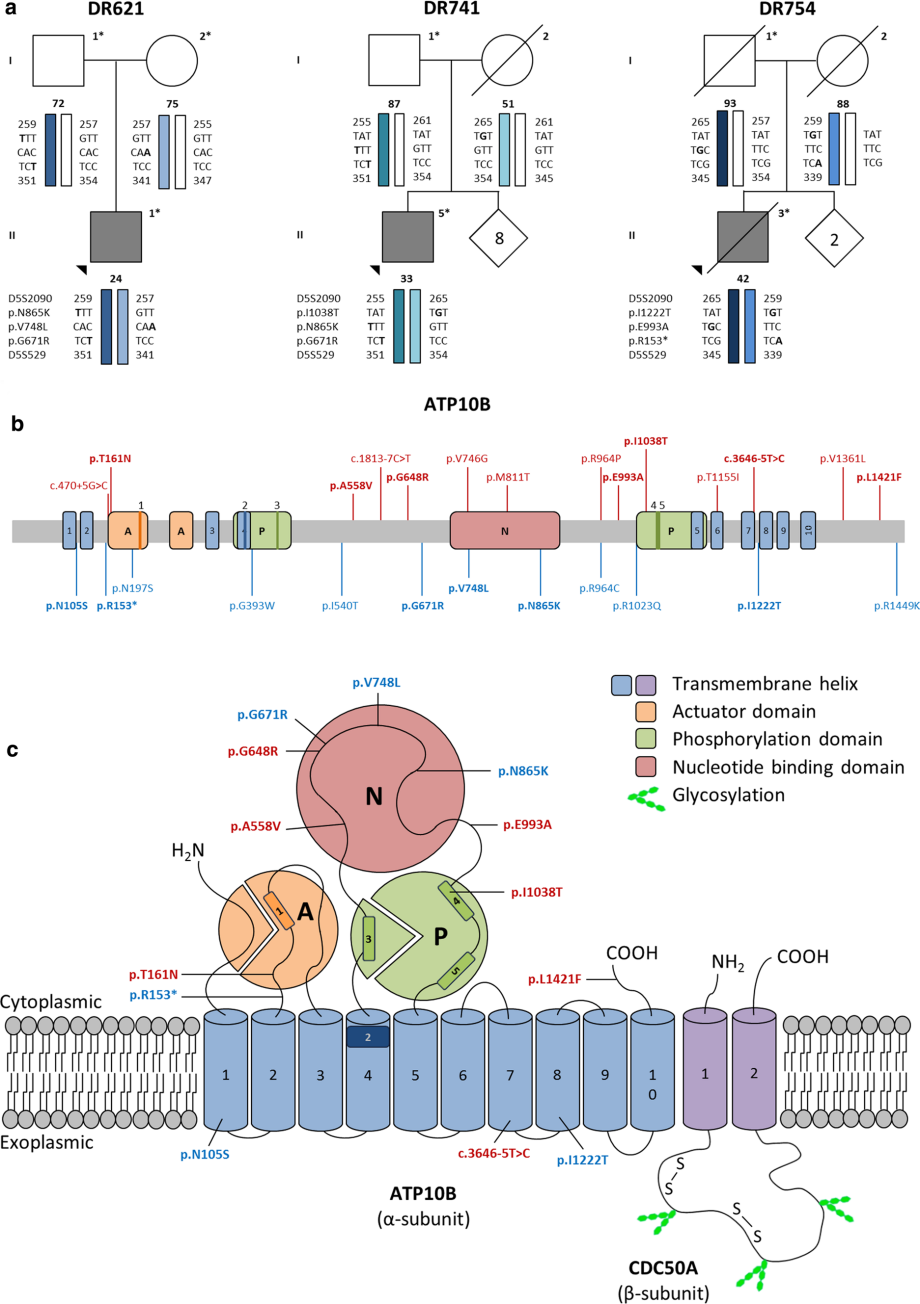
Genetic data of *ATP10B* in EOPD patients and the Belgian PD and DLB cohort. **a** Segregation data of *ATP10B* compound alleles in families of EOPD patients. DNA availability of relatives is indicated with an asterisk (*) and onset age of disease of patients and age at examination of unaffected relatives is indicated below the symbol. Numbers within each diamond represent unaffected at risk individuals included in the segregation analysis. Circles represent females; squares, males; filled symbols, patients; slash, deceased; arrowhead, index patient. **b** Linear representation of ATP10B with indication of all variants identified in PD and DLB patients*.* Domains based on the InterPro database [51], protein nomenclature according to NP_079429. Variants indicated in red are patient specific, variants indicated in blue were also found in control individuals, variants indicated in bold were found compound heterozygous in PD patients. **c** Schematic representation of ATP10B topology with indication of compound heterozygous variants in PD and DLB patients, and its interaction partner CDC50A, based on [8]. ATP10B motifs: 1, dephosphorylation site (^208^DGE); 2, substrate binding site (^380^PILS); 3, phosphorylation site (^433^DKT); 4 and 5, Mg^2+^ binding sites

WES data was generated for 52 unrelated EOPD patients with an AAO below 50 years and negative for mutations in the major PD genes. We identified compound heterozygous mutations in *ATP10B* in three EOPD patients DR621, DR741 and DR754 (Table 1; Table S1). Segregation analysis of family members of these patients provided evidence for a *trans* location of the mutant *ATP10B* alleles (Fig. 1a). Additionally, WGS data were obtained for patient DR621 and his parents, whom were neurologically healthy at ages of 75 and 72. Structural variants, including copy number variations, were excluded as a possible disease cause in the family. Candidate variant analysis of the WGS data identified eight candidate genes for PD, including *ATP10B*, but none with a de novo variant (Table S2). No rare or low-frequency variants (MAF < 5%) in line with the proposed inheritance models were identified in the seven other candidate genes after screening 120 EOPD patients. This case-unaffected-parents trio approach to search for novel candidate genes independently nominated *ATP10B* as a candidate gene for PD in DR621.

**Table 1:** PD and DLB carriers of compound heterozygous *ATP10B* mutations

Patient	Dx	Gender	AAO	∆CDS^a^	ΔAA^b^	MAF gnomAD (%)	MAF patient cohort (%) *n* = 843	MAF control cohort (%) *n* = 598	F compound heterozygotes in patients (%)	F compound heterozygotes in controls (%)	F compound heterozygotes expected^c^ (%)
DR621	PD	Male	24	c.2011G>A	p.G671R^e^	2.0	1.8	1.6	0.12	0.00	0.0018
				c.2595C>A	p.N865K^e^	2.0	1.8	1.6			
				c.2242G>T	p.V748L^f^	0.11	0.12	0.084			
DR741	PD	Male	33	c.2011G>A	p.G671R^e^	2.0	1.8	1.6	0.12	0.00	0.00060
				c.2595C>A	p.N865K^e^	2.0	1.8	1.6			
				c.3113T>C	p.I1038T^f^	–	0.059	0			
DR754^d^	PD	Male	42	c.457C>T	p.R153*^e^	0.49	0.18	0.25	0.12	0.00	0.000072
				c.3665T>C	p.I1222T^e^	1.2	1.3	1.1			
				c.2978A>C	p.E993A^f^	0.0047	0.059	0			
DR1046	PD	Male	68	c.482C>A	p.T161N	0.041	0.12	0	0.24	0.00	0.000048
				c.1942G>A	p.G648R	0.082	0.12	0			
DR1140	PD	Female	63	c.314A>G	p.N105S	0.30	0.059	0.42	0.12	0.00	0.000072
				c.1673C>T	p.A558V	0.00088	0.059	0			
DR1440	PD	Female	37	c.482C>A	p.T161N	0.041	0.12	0	0.24	0.00	0.000048
				c.1942G>A	p.G648R	0.082	0.12	0			
DR1504	DLB	Male	64	c.4261C>T	p.L1421F	0.0078	0.059	0	0.12	0.00	0.000024
				c.3646-5T>C	–	0.019	0.12	0			

Clinical symptoms of patients are summarized in Table S2

*Dx* diagnosis, *PD* Parkinson’s disease, *DLB* dementia with Lewy bodies, *∆CDS* coding sequence substitution, *ΔAA* amino acid substitution, *MAF* minor allele frequency, *F* frequency, *AAO* age at onset, *gnomAD* Genome Aggregation Database [60]

^a^Coding nomenclature according to NM_025153

^b^Protein nomenclature according to NP_079429

^c^Frequency calculated according to the Hardy–Weinberg principle, using the MAF of single alleles in the PD, DLB and control cohort (*n* = 1441)

^d^Deceased without autopsy

^e^Variants in *cis* configuration

^f^Variants on the other haplotype: indicating *trans* configuration

Targeted resequencing of all exons and splice site junctions of *ATP10B* in the Belgian cohorts of 617 PD and of 226 DLB patients, identified three PD carriers and one DLB carrier of compound heterozygous *ATP10B* mutations (Table 1). No prominent effect on splicing was predicted of the *ATP10B* c.3646-5T>C mutation in the DLB patient DR1504 (Table S3), though we were not able to assess the effect on mRNA splicing in the patient’s biomaterials because *ATP10B* mRNA expression is not detectable in blood (Fig. S2).

In total we observed in patients, 26 non-synonymous coding and splice site variants with a MAF < 5% in *ATP10B* (Fig. 1b) of which 14 variants were solely observed in patients (Fig. 1b, Table S4). The other 12 variants were present at low frequency (MAF < 5%) in a matched Belgian control cohort (Fig. S1). More common, but probably benign compound heterozygous combinations (frequency > 0.01%) were observed in both the patient and control cohorts (Table S7).

Before, ATP10B was identified as a P4-type transport ATPase of unknown function present in the late endo-/lysosomal compartment [73]. P4 ATPases are lipid flippases that use ATP to drive the transport of lipids from the lumen to the cytosolic membrane leaflet, establishing the vitally important lipid asymmetry between two membrane leaflets [8, 84]. We established that the mutant compound alleles in patients are mostly clustering around the catalytically nucleotide binding, phosphorylation, and actuator domains of ATP10B (Fig. 1c).

### *ATP10B* mRNA expression is reduced in idiopathic patients

We analyzed *ATP10B* mRNA levels in different tissue samples and observed expression in the gastrointestinal system and the brain (Fig. S2a). In the brain, *ATP10B* expression was enriched in regions of PD pathology [16], predominantly in the medulla oblongata, substantia nigra, as well as the basal ganglia (Fig. S2b). *ATP10B* mRNA expression was further assessed in brain material of autopsy confirmed PD or DLB patients and control individuals and showed in patients a significant reduction in both the medulla oblongata and the substantia nigra. (Fig. S2c, d).

### ATP10B is a late endo-/lysosomal GluCer/PC flippase

**Fig. 2:**
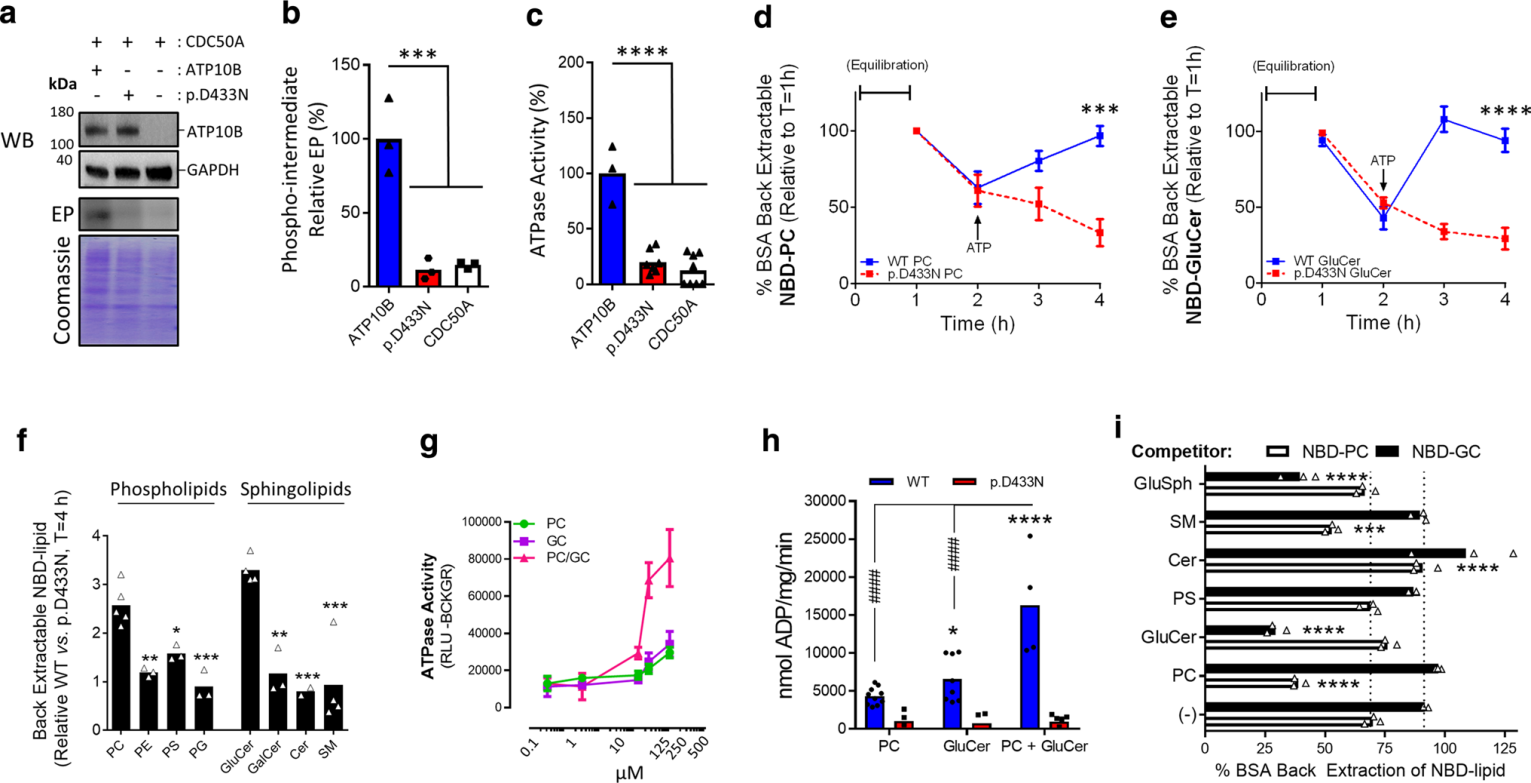
ATP10B mediates the translocation of phosphatidylcholine and glucosylceramide. Microsomes of HeLa cells with stable overexpression of CDC50A alone or in combination with either ATP10B WT or the catalytic p.D433N mutant were assessed for ATP10B expression and activity. Cells overexpressing CDC50A alone or co-expressing the catalytic mutant p.D433N were used as negative controls. **a** Upper part depicts a Western blot (WB) showing the comparable expression levels of ATP10B WT and p.D433N versus GAPDH as loading control. Lower part depicts a representative radiogram of the ^32P^-ATP based autophosphorylation experiment (EP, phospho-enzyme intermediate). The Coomassie gel staining was used as a loading control for the radiogram. **b** Bar graph summarizes the data of (**a**). **c** Differences in ATPase activity (ADP Glo assay) were investigated in ATP10B WT versus p.D433N or CDC50A-only membrane fractions. Reactions were initiated by the addition of 5 mM ATP and sustained for 30 min at 37 °C. Microsomes were assessed for their potential to translocate nitrobenzoxadiazole (NBD)-labeled phosphatidylcholine (NBD-PC, **d**) or NBD-glucosylceramide (NBD-GluCer, **e**) over time (0–4 h, 37 °C). We followed the lipid translocation by determining the fraction of fluorescent NBD-labeled lipids that is extractable from the accessible cytosolic membrane leaflet by fatty acid free bovine serum albumin (BSA). Following spontaneous translocation of fluorescently labeled lipids from the cytosolic to exoplasmic membrane leaflet (1–2 h), reactions were initiated by the addition of 1 mM ATP addition (at the 2 h time point). **f** Bar graph depicts the lipid specificity of ATP10B, showing that ATP10B preferentially translocates NBD-PC and NBD-GluCer (the bar graph summarizes the results of the translocation assay depicted in **d**, **e** and Fig. S3a–f). **g** Dose response curve for the stimulatory effects on the ATPase activity of ATP10B WT of the non-labeled lipids PC and GluCer, alone or in combination. **h** ATPase activity of microsomes was measured under the presence of a final concentration of 500 µM PC or GluCer, alone or in combination. **i** The NBD-PC/NBD-GluCer translocation potential of ATP10B was assessed alone or in combination with equimolar addition of non-fluorescent PC, GluCer, phosphatidylserine (PS), ceramide (Cer), sphingomyelin (SM) or glucosylsphingosine (GluSph) for 4 h at 37 °C. All data are the mean of three independent experiments. The immunoblots and autoradiograms are a representation of three independent experiments. Significance was determined by One Way ANOVA with either Dunnett’s (**b**, **c**, **f**, **i**) or Tukey’s (**h**) post hoc corrections. For time dependent lipid translocation assays (**d**, **e**), significance was assessed by Two Way ANOVA. In all cases *P < 0.05, **P < 0.01, ***P < 0.001 and ****P < 0.0001

The yeast orthologs of ATP10B, Dnf1p and Dnf2p, transport two classes of lipids, the phospholipid PC and the sphingolipid GluCer, whereas two close human isoforms, ATP10A and ATP10D, transport PC and GluCer respectively in the plasma membrane [73, 94]. Moreover, most mammalian P4-type ATPases, including ATP10A-D, depend on CDC50A forming a heteromeric complex that facilitates exit from the endoplasmic reticulum [8]. In HeLa cells the CDC50A isoform 1 (Accession NP_060717.1) was shown to guide human ATP10B to the late endo-/lysosomes [73]. Here, we confirmed the interaction between CDC50A isoform 1 and ATP10B in HEK293T cells by co-immunoprecipitation and split-luciferase protein complementation assays (Fig. S3a, b), and also observed a predominant late endo-/lysosomal localization of eGFP-labeled ATP10B (Accession O94823.2, longest isoform of 1461 amino acids) in HeLa cells co-expressing CDC50A (Fig. S4a, b). ATP10B contains typical hallmark motifs of other P4-type ATPases [12] (Figs. 1b, c, S1), and similar to other P4-type lipid flippases, ATP10B spontaneously forms a phospho-intermediate (Fig. 2a, b). The autophosphorylation and subsequent ATPase activities are fully abolished by mutating the phosphorylation acceptor residue p.D433 that is part of the catalytically important P-type ATPase signature motif ^433^DKTGTLT (Fig. 2a, c). The eGFP-labeled p.D433N mutant is also well-expressed in the endo-lysosomal compartments of HeLa cells (Fig. S4c, d), and due to its lack of enzymatic activity the p.D433N mutant serves as an important catalytic control for the functional assays to determine the biological transport activity of ATP10B, similar as in [111]. The p.D433N mutant is further used throughout this study as a loss-of-function control for the evaluation of the disease associated mutations.

Based on sequence comparison and phylogenetic relationship, we hypothesized that ATP10B may operate as a lipid flippase that exports GluCer and/or PC towards the outer membrane leaflet of the lysosome. Therefore, we performed a lipid translocation assay on isolated membrane fractions derived from HeLa cells stably overexpressing CDC50A with either ATP10B WT or p.D433N (Figs. 2d–f, S5) [73]. We followed the lipid translocation by determining the fraction of fluorescent NBD-labeled lipids that are extractable from the accessible cytosolic membrane leaflet by fatty acid free bovine serum albumin. We observed spontaneous translocation of fluorescently labeled lipids from the cytosolic to exoplasmic membrane leaflet. Following ATP addition, ATP10B WT, but not the catalytic dead mutant p.D433N, promoted NBD-GluCer and NBD-PC translocation from the exoplasmic to cytosolic membrane leaflet (Fig. 2d, e). ATP10B did not translocate NBD-phosphatidylethanolamine, NBD-phosphatidylserine, NBD-phosphatidylglycerol, NBD-galactosylceramide, NBD-ceramide, and NBD-sphingomyelin (Figs. 2f, S5). Unlabeled PC and GluCer also stimulated the ATPase activity of ATP10B WT, but not p.D433N (Fig. 2g, h), further confirming the lipid specificity, and indicating that the lipid dependent ATPase and translocation activities are coupled. Moreover, the co-incubation with PC and GluCer synergistically stimulated the ATPase activity of ATP10B more than PC or GluCer separately (at the same final lipid concentration), suggesting that the two lipids do not compete for the same substrate binding site. This was further verified by competition translocation assays demonstrating that equimolar concentrations of non-fluorescent GluCer and PC compete only with the translocation of the corresponding NBD-labeled analogs (Fig. 2i). Also, a modest inhibitory effect of GluSph on NBD-GluCer, and sphingomyelin on NBD-PC translocation was observed. Together, we demonstrated that ATP10B is a late endo-/lysosomal lipid flippase that translocates the lipids GluCer and PC towards the cytosolic membrane leaflet.

### Disease associated mutants impair functional activities of ATP10B

**Fig. 3:**
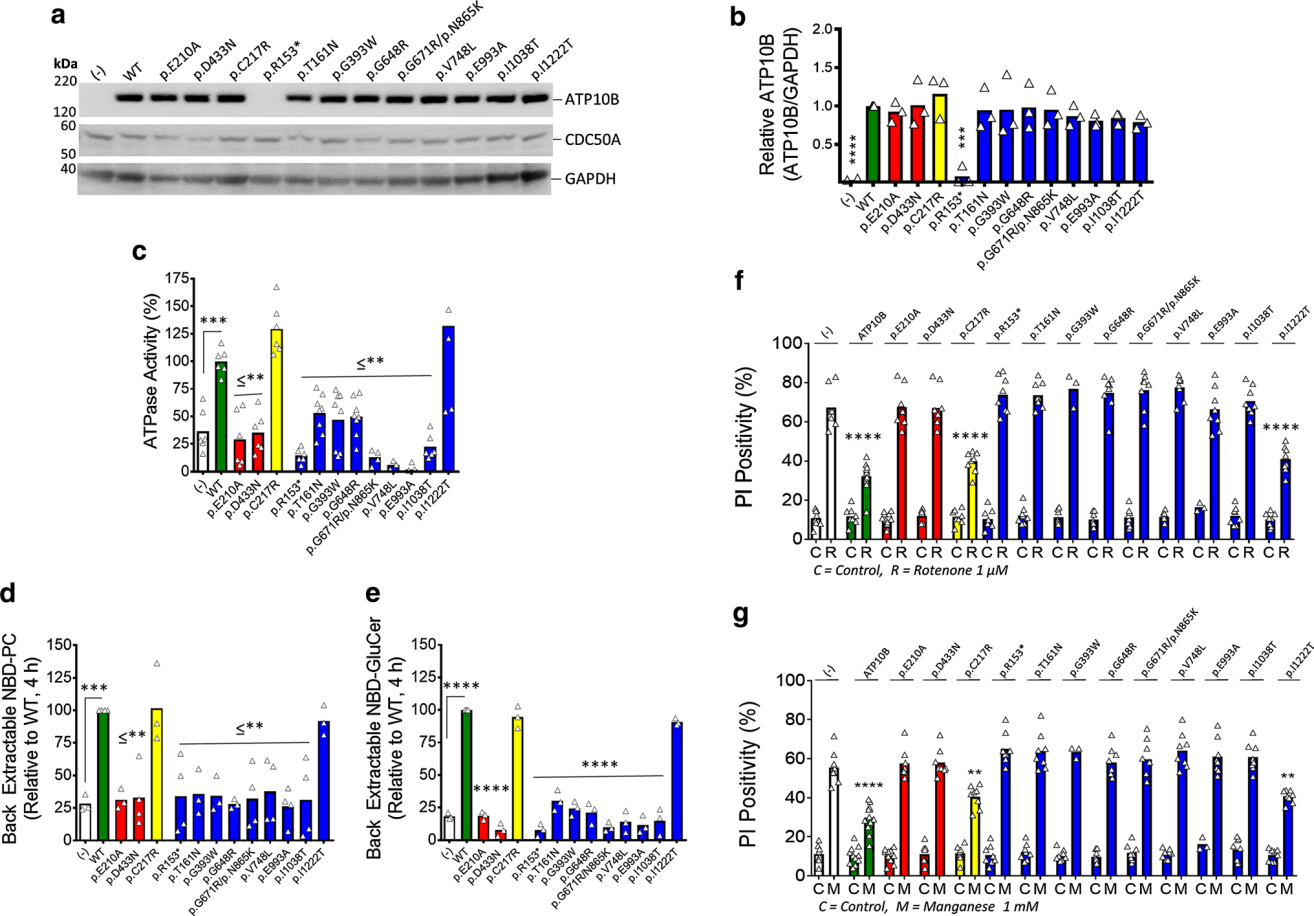
ATP10B disease mutations represent loss of function. **a** Protein expression of HeLa cells stably overexpressing flag-tagged CDC50A alone (–) or in combination with ATP10B WT, the catalytic variants p.E210A or p.D433N, the identified variants p.R153*, p.T161N, p.G393W, p.G648R, p.G671R/p.N865K, p.V748L, p.E993A, p.I1038T and p.I1222T or the polymorphism p.C217R. Immunoblotting was performed with primary antibodies against ATP10B, the CDC50A Flag-tag and GAPDH, which was used as a loading control. Immunoblots presented are representative of 3 independent observations. **b** All ATP10B variants are expressed to comparable protein levels, except for p.R153* (based on three independent blots for which the average ATP10B/GAPDH expression is depicted in the bar graph). **c** Microsomal fractions of the aforementioned stable cell lines were compared for their ATPase activity via an ADP Glo luciferase assay (normalized as % of WT ATP10B ATPase activity). Microsomes of HeLa cells expressing WT, clinical and control variants of ATP10B were compared for their capacity to translocate NBD-PC (**d**) or NBD-GluCer (**e**) at 4 h (37 °C) post ATP addition. Data are expressed as relative to the WT control. **f**, **g** PD-associated and catalytic ATP10B mutants fail to protect HeLa cells against rotenone and MnCl_2_ toxicity. The HeLa cell lines stably overexpressing CDC50A alone (–) or in conjunction with ATP10B, catalytic controls, clinical variants or the polymorphism were exposed to either rotenone (R, 1 µM, **f**) or MnCl_2_ (M, 1 mM, **g**) for 48 h, in comparison to DMSO control (C), prior to cell death analysis by propidium iodide (PI) based flow cytometry. All data are presented as the mean of three independent experiments ± SEM. Significance was assessed by one way ANOVA with Dunnett’s (to ATP10B WT) post hoc corrections, whereby **P < 0.01, ***P < 0.001 and ****P < 0.0001

We anticipated a loss of function mechanism similar to other autosomal recessive PD genes based on the identification of the premature stop codon mutation p.R153* in patient DR754. Moreover, all compound heterozygous missense mutations affect residues that are highly conserved across a diverse array of species (Fig. S6a), and are frequently positioned within conserved and functionally important domains of P-type ATPases (Fig. 1c). To investigate the effect of the compound heterozygous mutations in *ATP10B* identified in patients on the GluCer/PC translocation or ATPase activity, we transduced HeLa cells stably overexpressing CDC50A combined with ATP10B WT, two catalytic dead mutants p.D433N and p.E210A (mutation in dephosphorylation motif), mutants found compound heterozygous in patients (p.R153*, p.T161N, p.G393W, p.G648R, p.G671Rp.N865K, p.V748L, p.E993A, p.I1038T, p.I1222T) or a benign polymorphism (p.C217R, MAF 0.81). The resulting stable cell lines exhibited comparable ATP10B expression, except for the p.R153* truncation, which demonstrated a loss of protein expression when transfected into both HeLa and HEK293T cells (Figs. 3a, b, 6b). Moreover, full-length mutants retained their capacity to interact with CDC50A (Fig. S3c) highlighting similar interaction and trafficking as for wildtype ATP10B, allowing for an unbiased assessment of their functionality. Functional assessment demonstrated that all patient-associated mutants displayed impaired ATPase activity comparable to the catalytic dead mutants p.D433N and p.E210A (Fig. 3c). The reduced ATPase activity of the mutants correlated with an impaired NBD-GluCer and NBD-PC translocation activity (Fig. 3d, e), establishing a loss-of-function mechanism of the mutations compound heterozygous in patients. The loss of ATP10B function observed for the p.R153* mutant may be a consequence of the reduced expression levels in combination with the lack of catalytic domains. The p.I1222T mutant and the polymorphism p.C217R presented functional activities similar to WT ATP10B (Fig. 3c–e), indicating that p.I1222T is a benign variant. However, in patient DR754, this variant occurs in *cis* configuration with the damaging p.R153* mutation, while p.E993A, also presenting impaired functionality (Fig. 3c–e), is located in *trans* (Fig. 1a).

To investigate whether ATP10B exerts a cell protective effect, stable cell lines were exposed to the heavy metals MnCl_2_ and ZnCl_2_, the 26S proteasome inhibitor bortezomib and the mitochondrial complex I inhibitor rotenone, stressors that evoked a phenotype in cell models of ATP13A2 loss of function, another late endo-lysosomal P-type ATPase implicated in PD [26, 48, 68]. ATP10B WT significantly protected HeLa cells against rotenone and MnCl_2_, but not against ZnCl_2_ or Bortezomib (Fig. S7). Importantly, the disease-associated ATP10B mutants fail to provide cellular protection against rotenone and MnCl_2_ exposure, analogous to the catalytic dead mutants and overexpression of CDC50A without ATP10B, whereas the benign variants p.I1222T and p.C217R were able to provide cellular protection against both stress conditions to the same extent as WT ATP10B (Fig. 3f, g). Addition of the caspase inhibitor Zvad-fmk significantly blocked cell death, indicating that the observed cell death depends on apoptosis (Fig. S8).

### ATP10B knockdown results in lysosomal dysfunction

**Fig. 4:**
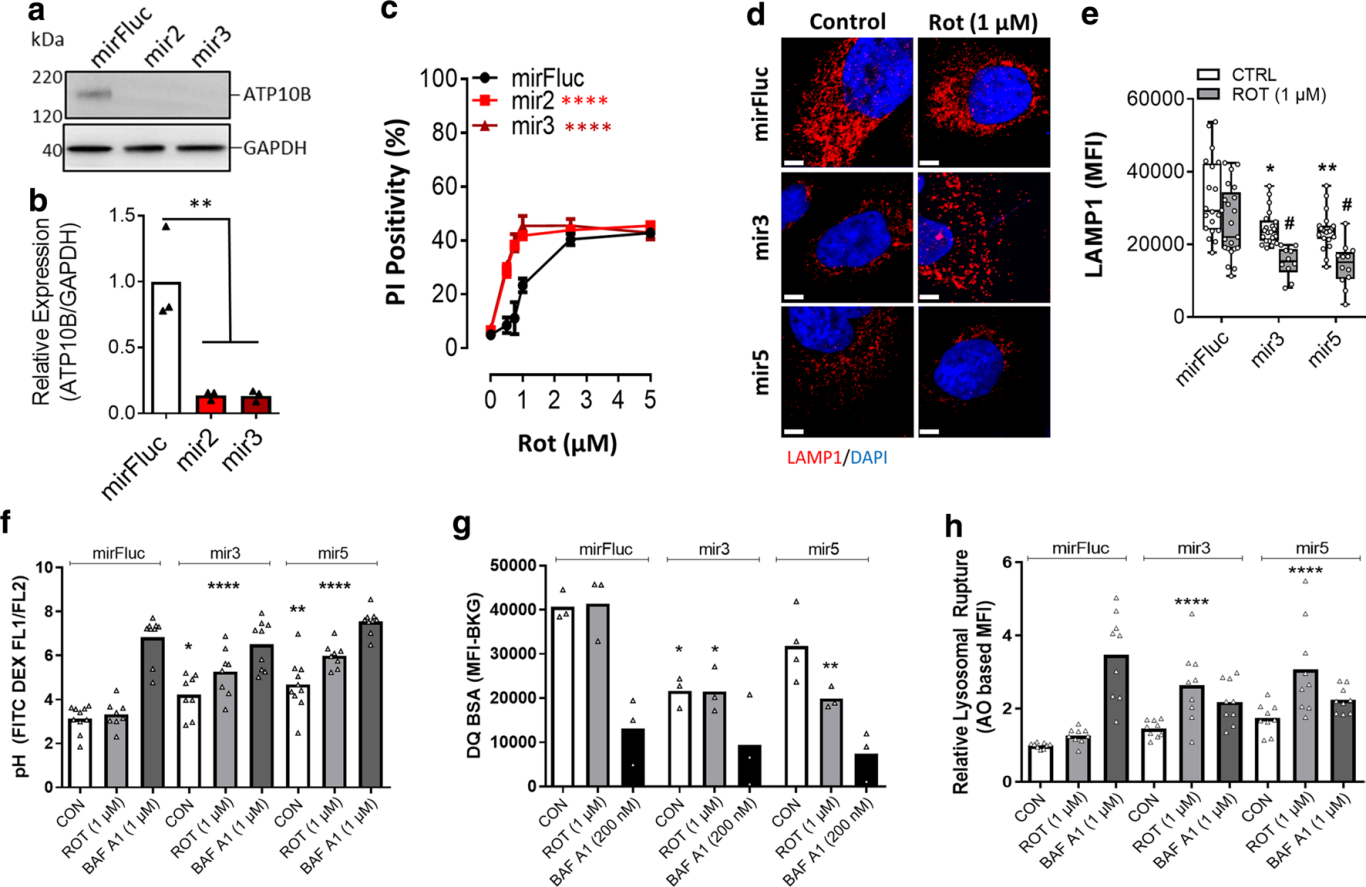
ATP10B knockdown sensitizes cells to rotenone and induces lysosomal dysfunction. **a**, **b** Immunoblot analysis confirms expression and knockdown of ATP10B protein in human WM-115 melanoma cells normalized to GAPDH as loading control. Two independent microRNA based short-hairpins (mir) were used to generate stable knockdown cells (mir2 and mir3). mirFluc serves as a control cell line that was transduced with a mir against the firefly luciferase gene (Fluc). Immunoblot is a representative example of 3 independent experiments with densitometry represented in a bar graph (b). **c** The effect of ATP10B knockdown was assessed on the sensitivity of WM-115 cells to increasing concentrations of rotenone (Rot). Propidium iodide (PI) exclusion (1 µg/ml, 5 min) based flow cytometry was used as a readout for cell death. **d–h** The effect of ATP10B knockdown on lysosomal functionality was assessed in the WM-115 mirFluc control cell line in parallel with two ATP10B knockdown models under basal and rotenone (1 µM, 24 h) conditions. **d** Lysosomal morphology was assessed by immunofluorescent staining to LAMP1. DAPI was used to highlight the nuclei. Scale bar 5 µm. **e** Images from (**d**) were captured by confocal microscopy, and mean fluorescence intensity (MFI) was depicted in a bar graph (**e**). **f** pH measurement based on FITC-dextran analysis. Cells were treated with 50 µg/ml FITC-dextran for 72 h. 24 h prior to the experiment rotenone (Rot, 1 µM) was added to the relevant conditions. Lysosomal pH was assessed ratio-metrically by flow cytometry and pH calculated by extrapolation from a pH standard curve generated from cells exposed with monensin containing pH defined buffers (pH 3–8). BAF A1 (200 nM), an inhibitor of the lysosomal proton pump, was used as a control to increase the pH. **g** Lysosomal degradative capacity was assessed by DQ-BSA, which following endocytic uptake is degraded in the lysosome leading to an increased fluorescence. Cells were exposed to 5 µg/ml DQ-BSA for 1 h prior to the treatment with either Rot or BAF A1 for a subsequent 4 h. General lysosomal proteolysis was then measured. **h** Lysosomal membrane integrity was assessed by an acridine orange based assay. Cells were treated with 5 µg/ml acridine orange (15 min, 37 °C), washed and treated with either Rot (1 µM) or BAF A1 (200 nM) for 4 h. Lysosomal membrane intactness was assessed by flow cytometry. Statistical validation was assessed by one way ANOVA with Dunnett’s post hoc and differences between cell line sensitivities were assessed by two way ANOVA’s whereby; *P < 0.05, **P < 0.01, ****P < 0.0001

To examine the consequences of ATP10B loss-of-function, we performed lentiviral based knockdown of ATP10B in two cell models presenting endogenous ATP10B expression. Extensive screening of cell lines, including neuroblastoma cell lines, indicated sufficient endogenous ATP10B expression in the human adenocarcinoma Im95m and melanoma WM-115 cells (Figs. 4, S9). To induce a loss-of-function phenotype, we generated for each cell line two independent knockdown models using mir-based short-hairpins that demonstrated a reduction of > 60% of ATP10B mRNA (Fig. S9a, b) and undetectable protein expression (Figs. 4a, b, S9d). Control cell models were generated with mirFluc, a mir against the firefly luciferase gene (Fluc). Knockdown of ATP10B significantly increased the sensitivity of the cells to rotenone and MnCl_2_ (Figs. 4c, S9c, g, h), but not to bortezomib or ZnCl_2_ (Fig. S9e, f, I, j), which was also observed in our mutant ATP10B overexpressing models (Fig. 3f, g), strengthening our conclusion that loss of ATP10B functionality increases cell toxicity towards PD-related insults.

To determine the underlying cause of cell death following loss of ATP10B we assessed lysosomal functionality in WM-115 cells (Fig. 4d–h). ATP10B knockdown cells presented a significant loss of lysosomal mass (Fig. 4d, e) and a higher lysosomal pH (Fig. 4f) resulting in a global reduction of lysosomal degradative capacity (Fig. 4g). A further increase in lysosomal pH was observed following rotenone exposure in the ATP10B knockdown cell models, but not in control cells (Fig. 4f). Moreover, rotenone exposure in ATP10B knockdown cells impaired lysosomal membrane integrity (Fig. 4h), which is a major driver of lysosome-dependent cell death [6].

### ATP10B knockdown sensitizes mouse cortical neurons to cell death

**Fig. 5:**
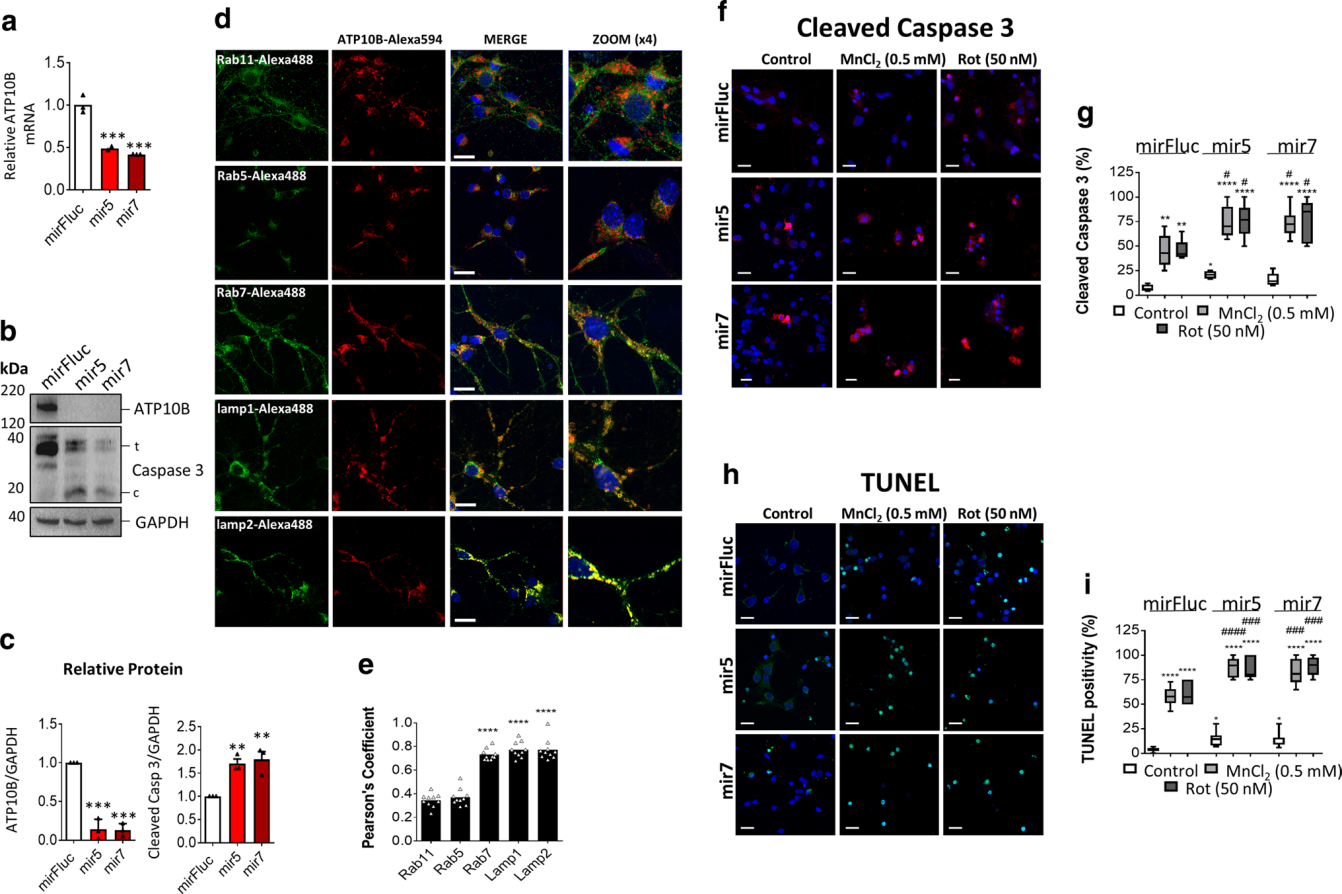
Loss of ATP10B expression sensitizes cortical neurons to PD-related stressors. **a–c** ATP10B expression in isolated murine cortical neurons and after knockdown with lentiviral shRNA transduction (mir5 and mir7) was assessed by qRT-PCR (**a**) and immunoblotting (**b**, **c**). ATP10B, as well as total (t) and cleaved (c) caspase 3 were probed via immunoblotting (**b**). GAPDH was used as a loading control. Bar graphs represent the densitometrical analysis of ATP10B and cleaved caspase 3 expression (**c**). **d**, **e** ATP10B is expressed in the late endo-/lysosomal compartment of isolated mouse cortical neurons. **d** ATP10B (red) co-localization with the endocytic compartments (green) was performed toward Rab11 (recycling endosomes), Rab5 (early endosomes), Rab7 (late endosomes), Lamp1 (late endo/lysosomes) or Lamp2 (lysosomes). DAPI was used as a marker of the nucleus. Scale 10 µm. **e** Pearson’s co-efficient for the co-localization probability of ATP10B with the aforementioned endocytic compartments. **f–i** ATP10B knockdown induces neuronal death, which is exacerbated by rotenone or manganese (MnCl_2_) treatment. ATP10B was knocked down (mir5, mir7) in cortical neurons, in comparison to control (mirFluc). Neurons were subsequently exposed to 50 nM rotenone or 0.5 mM MnCl_2_ (24 h) and cell death was assessed by cleaved caspase 3 antibody (**f**–**g**, red) or TUNEL (**h**–**i**, red) staining based confocal microscopy. DAPI (blue) was used as a marker of the nucleus. Scale bar 25 µm. Immunoblots are representative of three independent experiments, whereas quantification of the caspase 3/DAPI (**g**) or TUNEL/DAPI (**i**) ratio is presented in bar graphs and expressed in percentage. All data depict averages of a minimum of three independent experiments ± SEM. Significance was assessed by one-way ANOVA with either Dunnett’s post hoc (**e**, to Rab11; **a**, **c** to mirFluc) or Tukey’s (**g**, **i**) post hoc whereby, 1 mark *P* < 0.05, 2 marks *P* < 0.01, 3 marks *P* < 0.001, 4 marks *P* < 0.0001

**Fig. 6:**
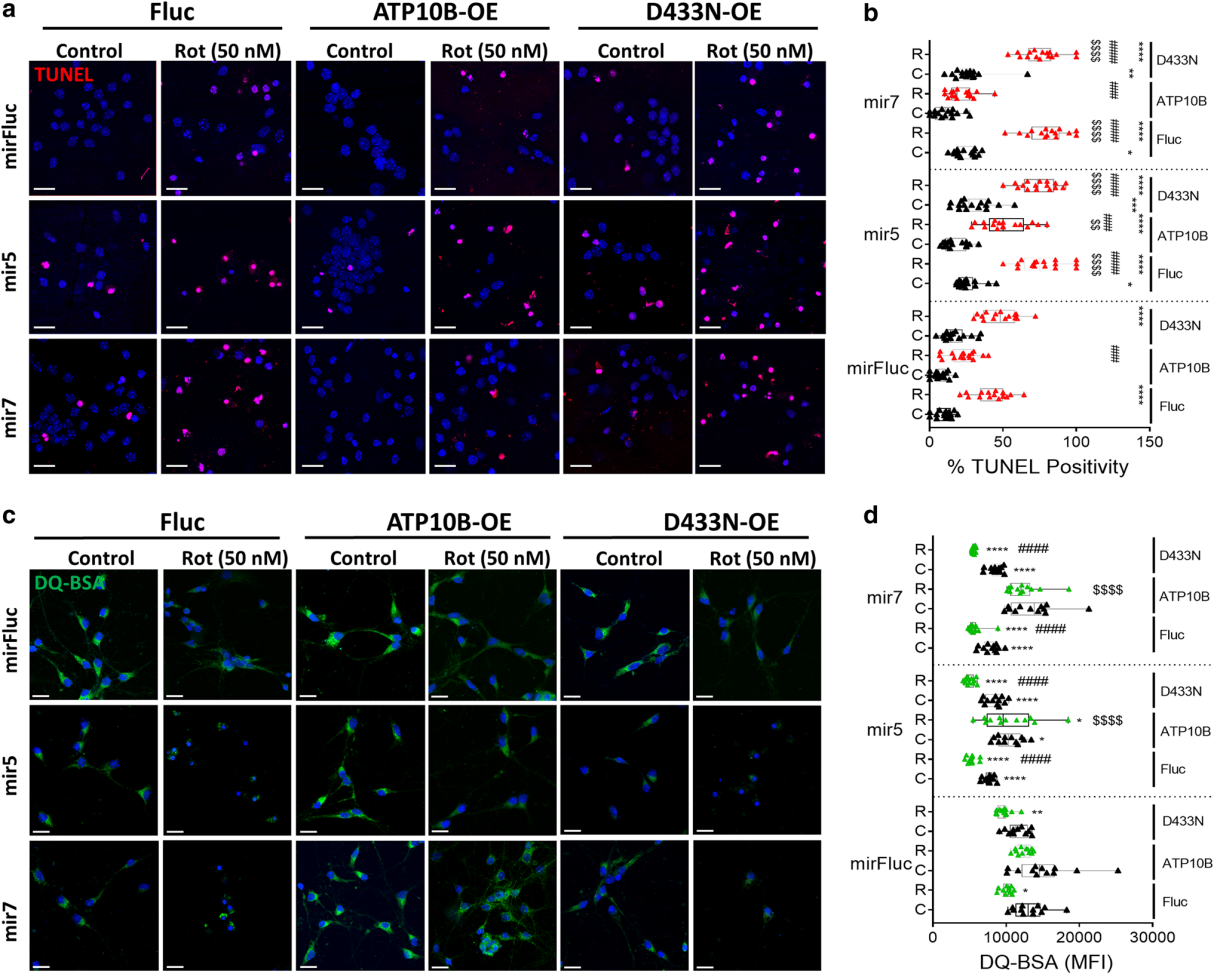
Increased neuronal sensitivity of ATP10B knockdown can be recovered by active ATP10B. **a**, **b** Control (mirFluc) and ATP10B knockdown (mir5 and mir7) cortical neurons were transduced with Fluc (Firefly luciferase), human WT ATP10B or p.D433N mutant. Following ATP10B recovery neurons were exposed to rotenone (Rot, 50 nM, 24 h) and sensitivity assessed by TUNEL (red) staining and confocal microscopy. DAPI (blue) was used as a reference. **b** Percentage of DAPI stained cells that were positive for TUNEL is expressed in a boxplot. **c**, **d** The capacity of ATP10B to recover lysosomal functionality under basal and rotenone (50 nM) conditions was assessed via the DQ-BSA assay (green 10 µg/ml). DAPI was used to visualize the nuclei (blue). **d** The mean fluorescent intensity of each condition in (**c**) was quantified and represented in a boxplot graph. White scale bar in cell images corresponds to 25 µm. All other data depict averages of a minimum of three independent experiments ± SEM. Statistical validation was assessed by one way ANOVA with Dunnett’s post hoc and differences between cell line sensitivities were assessed by two way ANOVA’s whereby; * versus control mirFluc (Fluc), $ versus rotenone in the mirFluc, and # is cellular control. 1 mark *P* < 0.05, 2 marks *P* < 0.01, 3 marks *P* < 0.001, 4 marks *P* < 0.0001

We knocked down ATP10B in isolated mouse cortical neurons to establish the link between loss of ATP10B and neurodegeneration. Before knockdown, endogenous ATP10B expression in isolated mouse cortical neurons was verified by qRT-PCR and immunoblotting (Fig. 5a–c). Moreover, we confirmed the localization of ATP10B in CD63/LAMP1-positive compartments via immunocytochemistry, demonstrating that also in neurons endogenous ATP10B is confined to the late endo-lysosomes (Fig. 5d, e). Post isolation, ATP10B was knocked down in primary neurons via lentiviral shRNA transduction, which significantly reduced ATP10B expression (Fig. 5a–c) and correlated with a significant increase in cleaved caspase 3 expression. Control mirFluc primary neurons were generated for comparison. To evaluate the protective effect of ATP10B in neurons, we first determined a dose response curve for rotenone and MnCl_2_ toxicity and identified 50 nM rotenone and 0.5 mM MnCl_2_ as potent inducers of caspase 3 induced cell death in the isolated neurons (24 h, Fig. S10). Importantly, ATP10B knockdown already sensitized neurons to cell death in basal conditions, as demonstrated by the cleaved caspase 3 and the TUNEL assay (Fig. 5b, c). Following exposure to either rotenone or MnCl_2_, ATP10B knockdown neurons were even more susceptible to cell death (Fig. 5f–i). This phenotype could be rescued by ectopic expression of human WT ATP10B, but not by ectopic expression of the ATP10B p.D433N mutant (Figs. 6a, b, S10c, d), providing evidence that ATP10B activity is coupled to neuronal survival. Finally, ATP10B knockdown significantly reduced the degradative capacity of the lysosomes in the neurons (Fig. 6c, d), which could also be rescued by the ectopic expression of human WT ATP10B, but not by expression of the p.D433N mutant of ATP10B.

## Discussion

In our Belgian cohorts, we identified six PD patients (6/617; 0.97%) and one patient clinically diagnosed with probable DLB (1/226; 0.44%), carrying compound heterozygous mutant alleles in *ATP10B* (Table 1). All 6 PD patients presented with a typical clinical PD phenotype, and two PD patients, DR741 and DR1140, developed cognitive dysfunction later in their disease course (Table S1). In the DLB cohort the frequency of *ATP10B* mutation carriers might be underestimated since obtaining an accurate clinical diagnosis of DLB is not obvious. A systematic review and meta-analysis of the accuracy of the clinical diagnosis revealed that about 20% of DLB patients have an incorrect diagnosis, in most cases AD [93]. If we maintained in our DLB cohort only the patients with a clinical diagnosis of probable DLB (*n* = 113) or a neuropathological diagnosis of DLB (*n* = 70), the frequency of the compound heterozygous mutation carriers increased to 0.55%. All patient carriers of compound heterozygous *ATP10B* mutations tested negative for mutations in major genes linked to different neurodegenerative brain diseases (Table S5). In the control cohort, we found two carriers of compound heterozygous *ATP10B* mutant alleles (2/597; 0.33%) (Table S6).

The compound mutant allele combinations in patients were rare (frequency < 0.01%) and specific for the patient cohort. Among the patient carriers the AAO varied from 24 to 68 years, and in the two control carriers the AAI was 69 and 75. The high variability in AAO and the presence of compound heterozygous mutant carriers in the control group is probable due to variable effects of single mutant alleles on *ATP10B *expression and their different combinations in compound carriers. Variable penetrance likely leads to variable expression and functioning of ATP10B as shown in the functional in vitro studies (Fig. 3c–e). Incomplete penetrance of genetic alleles was also observed in *LRRK2* and *GBA1* mutation carriers [9, 67, 101].

The observation of six compound PD carriers versus two compound control carriers is suggestive of an enrichment of compound heterozygous mutations in patients. However, the statistical analysis was not significant (*p* = 0.2879), most likely because of the small numbers in the Belgian cohorts. An estimation of the number of subjects needed for an adequate study power, taking into account the low frequency of compound heterozygous carriers, showed that a minimum of 5000 subjects (~ 2500 PD patients and ~ 2500 control individuals) would be required to reach 80% power.

Overall our results indicate that recessive mutations in *ATP10B* are associated with increased risk for PD, which is different from heterozygous dominant mutations in other PD risk genes. This might explain why *ATP10B* was not identified in large-scale PD GWAS studies using an allelic model [74]. Notably, homozygosity mapping and exome sequencing in 62 isolated individuals with early-onset parkinsonism and confirmed consanguinity, identified *VPS13C* as a candidate gene for autosomal recessive PD [61]. The use of a validation cohort [61], and an independent replication cohort confirmed a role for *VPS13C* in autosomal recessive PD [24, 97]. Another whole exome sequencing study in 50 early-onset patients with PD has nominated interesting variants in *SPG7* [106]. Replication in exome data from 1148 European PD patients revealed significant association of heterozygous of p.A510V in *SPG7* with PD risk [106]. Another independent study reported that parkinsonism is frequently observed in spastic paraplegia patients with pathogenic variants in *SPG7* [25]. These examples demonstrated that despite the limitations of small discovery cohorts, valuable data can be obtained through replication in independent and larger cohorts. Anyway, replication of our findings in independent and larger cohorts is needed to provide statistical association data allowing to estimate the frequency of *ATP10B* compound heterozygous PD and DLB carriers [11, 53, 92].

*ATP10B* mRNA is mainly expressed in the gastrointestinal track and the brain. Interestingly, Lewy body pathology is also observed in the enteric nervous system of PD patients [18, 115] and the majority of PD patients develop eventually gastrointestinal disorders such as dysphagia, constipation and gastroesophageal reflux. The incidence of gastrointestinal disorders in PD patients increases over time and reaches approximately 65% four years after PD diagnosis [66]. We observed a reduction in *ATP10B* mRNA expression in the medulla oblongata and the substantia nigra of idiopathic patients compared to control individuals, which could result in reduced ATP10B functionality and might, at least partially, drive pathology. Otherwise, *ATP10B* expression might become downregulated in response to PD-related pathological insults.

Our biochemical results confirm that ATP10B functionally belongs to the ATP10A/D sub-class of human lipid flippase isoforms that share highly conserved functional domains for GluCer and PC transport, pointing to a physiological role of the ATP10A, B and D transporters in GluCer/PC uptake or subcellular redistribution [94]. Interestingly, we here established that ATP10B transports both GluCer and PC synergistically via a non-competing translocation mechanism, suggesting distinct substrate binding sites and translocation pathways. Mutations in the yeast Dnf1/2 and human ATP10A/D isoforms were able to separate the GluCer/PC substrate specificity, indicating that GlcCer and PC are coordinated differently and may indeed be transported by independent pathways [94]. The results from our competition assay indicate that besides GluCer and PC, also GluSph and sphingomyelin may be transported by ATP10B. This can be rationalized by similarities in the lipid head groups of the transported lipid species, which is a major determinant of substrate specificity in lipid flippases [8, 84]. Further studies are needed to pinpoint more precisely which (combination) of the transported lipids of ATP10B may be responsible for the observed neuroprotective effect.

ATP10B demonstrates a cell protective effect towards the pesticide rotenone and heavy metal MnCl_2_, two agents that represent independent modes of action and that are considered as environmental risk factors for PD. Interestingly, like ATP10B, ATP13A2 (PARK9) is another late endo-/lysosomal P-type ATPase that is genetically linked to PD [28, 90]. As a lysosomal polyamine exporter, ATP13A2 provides protection to rotenone and MnCl_2_ [68, 111, 112], and also maintains lysosomal functionality and membrane integrity [111], pointing to a remarkable synergy between the two lysosomal export systems. Rotenone is a mitochondrial complex I inhibitor, whereas MnCl_2_ may inhibit the mitochondrial respiratory chain at the level of complex II [43, 102]. By redistributing lipids within the cell and/or by maintaining lysosomal functionality and membrane integrity, ATP10B may provide protection to mitochondrial dysfunction or oxidative stress.

Alterations in sphingolipid lipid homeostasis are linked to lysosomal dysfunction and involved in PD pathogenesis [65, 78, 87]. Heterozygous mutations in *GBA1* are associated with an increased risk for PD [30, 99]. Moreover, both α-synuclein and the phospholipase PLA2G6, the latter implicated in neurodegeneration with brain iron accumulation and early-onset dystonia parkinsonism [52, 56], have been associated with changes in lysosomal ceramide levels, though the exact mechanisms are still unknown [57, 64, 69]. Additionally, compelling associations between PD and genes involved in or linked to sphingolipid metabolism are emerging from genetic studies, including *SMPD1*, *GALC* and *SCARB2* [7, 21, 39]. Here, we identified *ATP10B* as a novel putative PD risk gene and key player in lysosomal functioning and sphingolipid metabolism. As a lysosomal GluCer and possible GluSph exporter, ATP10B may be considered as a direct regulator of the lysosomal GluCer/GluSph content, similar as GCase, which hydrolyzes glucose moieties from both GluCer and GluSph within lysosomes [30]. ATP10B may therefore synergize with GCase to maintain low levels of GluCer or GluSph in the lysosome in a subset of tissues, including the brain, where both ATP10B and GCase expression is observed. Similar to *ATP10B* mutations, *GBA1* mutations lead to a reduced or loss of function, which in turn results in a disturbed lysosomal GluCer/GluSph homeostasis and lysosomal dysfunction [37, 57]. Changes in GluCer levels may affect the V-Type ATPase activity at the lysosome [109], whereby loss of GluCer hinders compartment acidification, or alternatively accumulation of GluCer or its breakdown products may directly affect lysosomal membrane integrity. Loss of GCase function and elevated GluCer levels were also shown to increase α-synuclein aggregation, mitochondrial impairment, inflammation, and endoplasmic reticulum stress, though the exact mechanism is unclear [30, 57, 69, 100, 104]. Elevated lysosomal GluCer levels may be a common consequence of both dysfunctional ATP10B and GCase. GluCer reduction therapy by pharmacological inhibition of GluCer synthase may therefore be considered in PD treatment, which is currently in clinical trials for GBA1-associated PD [96]. We further propose that increasing ATP10B functionality for lysosomal substrate reduction therapy may be an attractive strategy to prevent lysosomal GluCer accumulation.

ATP10B dysfunction also diminishes lysosomal PC export, which may further contribute to the disease mechanism. Indeed, the PC lipid composition was shown to be disturbed in the substantia nigra of a PD rat model treated with 6-hydroxydopamine [36]. Moreover, loss of function mutations in *PLA2G6*, encoding a phospholipase involved in repair of oxidative damage to membrane phospholipids, membrane remodeling and iron homeostasis, are also shown to affect neuronal lipid homeostasis [14, 83, 98]. Loss of ATP10B activity increases neuronal stress in basal conditions, which is exacerbated, and results in enhanced cell death, in the presence of rotenone or MnCl_2_, two environmental risk factors of PD [38]. Lysosomal PC and/or GluCer transport may be relevant during oxidative stress to replace damaged lipids and provide membrane integrity, or by regulating lysosomal membrane dynamics during vesicular budding or fusion. To prevent toxicity and cell death, neurons heavily rely on the endo-/lysosomal system for the efficient removal of damaged mitochondria and toxic proteins, such as α-synuclein, using cell-autonomous (*e.g.* autophagy) and cell non-autonomous (*e.g.* exosome export) pathways [3]. Lysosomal dysfunction is one of the key players in PD pathology, therefore strategies aiming at improving lysosomal health and functionality may be considered for PD treatment [70].

In conclusion, we identified *ATP10B* as a PD risk gene and functionally characterized ATP10B as a late endo-/lysosomal GluCer/PC flippase that regulates lysosomal functionality and provides neuroprotection. All disease-associated compound heterozygous *ATP10B* mutant alleles presented impaired functional activities of ATP10B, suggesting that reduced lysosomal GluCer/PC export may increase PD risk. As even risk alleles with moderate effect sizes are beneficial to consider new therapeutic strategies [54], we provide a new potential target to tackle PD.

## Electronic supplementary material

Below is the link to the electronic supplementary material.

Supplementary file1 (PDF 1057 kb)

## Data Availability

The datasets generated during the current study are available from the corresponding author on reasonable request.
