# Supplementary material for: Mutated ATP10B increases Parkinson’s disease risk by compromising lysosomal glucosylceramide export
Source: Acta Neuropathol. 2020 Mar 14;139(6):1001–24. doi: 10.1007/s00401-020-02145-7 (PMC7244618; doi:10.1007/s00401-020-02145-7)
Supplement: Supplementary file 1 — Supplementary file1 (PDF 1057 kb) [file 401_2020_2145_MOESM1_ESM.pdf]

Mutated ATP10B increases Parkinson's disease risk by compromising lysosomal glucosylceramide export.

Shaun Martin[1]*, Stefanie Smolders[2,3,4]*, Chris Van den Haute[5,6], Bavo Heeman[2,3,4], Sarah van Veen[1], David Crosiers[2,3,7], Igor Beletchi[1], Aline Verstraeten[2,3,4], Helena Gossye[2,3,4,7,8], Géraldine Gelders[5], Philippe Pals[3,7], Norin Hamouda[1], Sebastiaan Engelborghs[3,8], Jean-Jacques Martin[3], Jan Eggermont[1], Peter Paul De Deyn[3,8], Patrick Cras[3,7], Veerle Baekelandt[5,6], Peter Vangheluwe[1#], Christine Van Broeckhoven[2,3,4#] and the BELNEU consortium.

**These shared first authors and [#]shared last and corresponding authors have contributed equally.*

[1]Laboratory of Cellular Transport Systems, Department of Cellular and Molecular Medicine, KU Leuven, Leuven, Belgium

[2]Center for Molecular Neurology, VIB, Antwerp, Belgium

[3]Institute Born-Bunge, Antwerp, Belgium

[4]University of Antwerp, Antwerp, Belgium

[5]Laboratory for Neurobiology and Gene Therapy, KU Leuven, Leuven, Belgium

[6]Leuven Viral Vector Core, KU Leuven, Leuven, Belgium

[7]Department of Neurology, Antwerp University Hospital, Edegem, Belgium

[8]Department of Neurology and Memory Clinic, Antwerp Hospital Network, General Hospitals Middelheim and Hoge Beuken, Antwerp, Belgium

Collaborators: The following members of the Belgian Neurology (BELNEU) consortium have contributed to this study by sampling and clinical characterizing PD patients included in the Belgian PD cohort: Chris van der Linden (Department of Neurology, General Hospital St. Lucas Ghent, Ghent, Belgium), Emke Maréchal (Department of Neurology and Memory Clinic,

Antwerp Hospital Network, General Hospitals Middelheim and Hoge Beuken, Antwerp, Belgium), Patrick Santens (Department of Neurology, University Hospital Ghent and University of Ghent, Ghent, Belgium), Wim Vandenberghe (Department of Neurology, University Hospitals Leuven, Leuven, Belgium), Bruno Bergmans (Department of Neurology and Center for Cognitive Disorders, General Hospital Sint-Jan Brugge, Brugge, Belgium).

Corresponding authors:

Prof. Christine Van Broeckhoven PhD DSc

VIB Center for Molecular Neurology,

University Antwerpen – CDE, Universiteitsplein 1, 2610 Antwerpen, Belgium

Tel. +32 3 265 1101; E-mail: christine.vanbroeckhoven@molgen.vib.ua.be

Prof. Peter Vangheluwe PhD

Department of Cellular and Molecular Medicine

KU Leuven Campus Gasthuisberg, O&N I Herestraat 49 - bus 802, 3000 Leuven

Tel. +32 16 33 07 20; E-mail: peter.vangheluwe@kuleuven.be

SUPPLEMENTARY TABLES AND FIGURES

Table S1. Clinical characteristics of *ATP10B* compound heterozygous PD and DLB carriers

Patient	Clinical diagnosis	AAO	AAE	Brady-kinesia	Rigidity	Tremor	Postural instability	Cognitive deterioration	Motor fluctuations	Dyskinesia	Current medication
DR621	PD	24	60	Yes	Yes	No	Yes	No	Yes	Yes	Levodopa/ carbidopa/ entacapone, rasagiline
DR741	PD	33	51	Yes	Yes	No	Yes	Yes	Yes	Yes	Levodopa/ carbidopa/ entacapone, rasagiline, amantadine
DR754	PD	42	56	Yes	Yes	Yes	Yes	No	Yes	No	Not available
DR1046	PD	68	50	Yes	Yes	No	Yes	No	Yes	No	Levodopa/ benserazide, rasagiline
DR1140	PD	63	75	Yes	Yes	No	Yes	Yes	Yes	Yes	Levodopa/ carbidopa/ entacapone, pramipexol
DR1440	PD	37	82	Yes	Yes	Yes	Yes	No	Yes	Yes	Levodopa/ carbidopa/ entacapone, rasagiline, pramipexol
DR1504	DLB	64	67	Yes	Yes	Yes	Yes	Yes	Yes	No	Levodopa/ benserazide

Note: All compound heterozygous *ATP10B* mutation carriers had no documented family history of PD. Abbreviations: PD, Parkinson’s disease; DLB, dementia with Lewy bodies; AAO, age at onset; AAE, age at last neurological examination, N.A., not available.

Table S2. Candidate gene mutations identified in whole genome sequencing data of family DR621

Gene	Inheritance	ΔCDS	ΔAA	MAF Belgian control cohort (%)	MAF gnomAD (%)
BCAN	Homozygous	c.2119C>A	p.L595I	1.6	0.9
ANKLE1	Homozygous	c.1078G>C	p.E268Q	1.4	1.3
ATP10B	Compound heterozygous	c.2011G>A	p.G671R	2.2	1.6
		c.2242G>T	p.V748L	0.1	0.1
		c.2595C>A	p.N865K	2.2	1.6
PSME1	Compound heterozygous	c.149A>G	p.N50S	0.5	0.1
		c.704G>A	p.R235K	0.2	0.2
ZNF323	Compound heterozygous	c.582G>A	p.R61Q	0.3	0.6
		c.1449G>A	p.R350H	0.1	0.0
ABCC12	Compound heterozygous	c.2549T>C	p.V850A	0.1	0.1
		c.3994-7C>T	-	0.4	0.2
TMSB15B	X-linked	c.253A>G	p.T44A	-*	-
LOC653354	X-linked	c.134C>A	p.A45E	-*	NA

Note: Abbreviations: An asterisk indicates that only male control individuals were taken into account to determine the MAF in the Belgian control cohort. ΔCDS, coding sequence substitution; ΔAA, amino acid substitution; MAF, minor allele frequency; gnomAD, Genome Aggregation Database [4]; NA, not applicable.

Table S3: In-silico predictions on mRNA splicing of *ATP10B* c.3646-5T>C

**Splicing prediction method**	**Reference sequence**	**Mutated sequence**
**[Range], Threshold**	**c.3646-5T**	**c.3646-5T>C**
SSF [0-100], ≥ 70	91.14	87.60 (-3.9%)
MaxEnt [0-12], ≥ 0	12.21	12.88 (+5.5%)
NNSPLICE [0-1], ≥ 0.4	0.99	0.99 (+0.0%)
GeneSplicer [0-24], ≥ 0	9.99	10.58 (+5.8%)

Note: Predicted effect on splicing due to *ATP10B* c.3646-5T>C, according to four different splicing prediction tools integrated in the Alamut Visual version v.2.11.0 (Interactive Biosoftware, Rouen, France): SpliceSiteFinder-like (SSF), MaxEntScan (MaxEnt), NNSPLICE and GeneSplicer. Splicing scores resulting from the reference and mutated sequences are listed within the complete range of possible values for each method. Only values passing the method-specific threshold are reported. The difference between the reference sequence score and the mutated sequence score are given in percentage.

Table S4. *ATP10B* variants identified in the PD, DLB and control cohorts

ΔCDS[a]	ΔAA[b]	MAF gnomAD (%)	MAF PD patients (%) n=617	MAF DLB patients (%) n=226	MAF control individuals (%) n=598	MAF PD, DLB and control cohort (%) n=1441
c.127A>T	p.T43S	0.0026	0	0	0.084	0.035
c.314A>G	p.N105S	0.30	0.081	0	0.42	0.21
c.409_419del	p.D137Tfs*3	-	0	0	0.084	0.035
c.457C>T	p.R153*	0.49	0.081	0.44	0.25	0.21
**c.470+5G>C**	**-**	**-**	**0.081**	**0**	**0**	**0.035**
**c.482C>A**	**p.T161N**	**0.041**	**0.16**	**0**	**0**	**0.069**
c.570_572del	p.L191del	0.00088	0	0	0.084	0.035
c.590A>G	p.N197S	0.070	0.24	0.44	0.084	0.21
c.961A>G	p.I321V	0.0	0	0	0.084	0.035
c.1177G>T	p.G393W	1.87	2.1	1.1	2.5	2.1
c.1559G>A	p.R520Q	0.0035	0	0	0.084	0.035
c.1619T>C	p.I540T	0.066	0.081	0.22	0.084	0.10
**c.1673C>T**	**p.A558V**	**0.00088**	**0.081**	**0**	**0**	**0.035**
**c.1813-7C>T**	**-**	**0.0**	**0**	**0.22**	**0**	**0.035**
**c.1942G>A**	**p.G648R**	**0.081**	**0.16**	**0**	**0**	**0.069**
c.1987A>G	p.R663G	-	0	0	0.084	0.035
c.2011G>A	p.G671R	2.0	1.9	1.8	1.6	1.7
**c.2237T>G**	**p.V746G**	**0**	**0.081**	**0**	**0**	**0.035**
c.2242G>T	p.V748L	0.11	0.081	0.22	0.084	0.10
**c.2432T>C**	**p.M811T**	**-**	**0.081**	**0**	**0**	**0.035**
c.2567G>A	p.R856Q	0.0047	0	0	0.084	0.035
c.2595C>A	p.N865K	2.0	1.9	1.8	1.6	1.7
c.2714G>C	p.R905P	0.0027	0	0	0.084	0.035
c.2890C>T	p.R964C	0.18	0.081	0	0.084	0.069
**c.2891G>C**	**p.R964P**	**0.039**	**0.081**	**0**	**0**	**0.035**
**c.2978A>C**	**p.E993A**	**0.0047**	**0.081**	**0**	**0**	**0.035**
c.3068G>A	p.R1023Q	0.10	0.081	0.22	0.17	0.14
c.3086G>A	p.R1029H	0.039	0	0	0.17	0.069

**c.3113T>C**	**p.I1038T**	**-**	**0.081**	**0**	**0**	**0.035**
**c.3464C>T**	**p.T1155I**	**-**	**0**	**0.22**	**0**	**0.035**
**c.3646-5T>C**	**-**	**0.019**	**0.081**	**0.22**	**0**	**0.069**
c.3665T>C	p.I1222T	1.2	1.2	1.6	1.09	1.2
**c.4081G>T**	**p.V1361L**	**0.00089**	**0.081**	**0**	**0**	**0.035**
**c.4261C>T**	**p.L1421F**	**0.0078**	**0**	**0.22**	**0**	**0.035**
c.4346G>A	p.R1449K	0.035	0.081	0.22	0.17	0.14

Note: Targeted resequencing of *ATP10B* in the patient and control cohorts identified 35 non-synonymous coding and splice site variants with a MAF < 5%. Patient specific variants are represented in bold. [a]Coding nomenclature according to NM_025153; [b]Protein nomenclature according to NP_079429. Abbreviations: ΔCDS, coding sequence substitution; ΔAA, amino acid substitution; MAF, minor allele frequency; gnomAD, Genome Aggregation Database [4]; PD, Parkinson disease.

Table S5. Major genes associated with neurodegenerative brain diseases.

**PD**	**AD**	**FTD**	**ALS**	**Prion disease**
LRRK2<br>*SNCA*<br>*PARK2*<br>*PINK1*<br>*PARK7*<br>*VPS13C*<br>*VPS35*<br>*EIF4G1*<br>*FBXO7*<br>*ATP13A2*<br>*GBA*<br>*AMBRA1*	*APP*<br>*PSEN1*<br>*PSEN2*<br>*APOE*<br>*CLU*<br>*CR1*<br>*PICALM*<br>*BIN1*<br>*CSF1R*	*GRN*<br>*C9orf72*<br>*MAPT*<br>*VCP*<br>*CHMP2B*	*TARDBP*<br>*FUS*<br>*SOD1*<br>*ELP3*	*PRNP*

Note: The complete coding region and intron/exon boundaries of the major genes associated with neurodegenerative brain diseases are included in a custom-designed gene panel (MASTR technology, Agilent, Multiplicom, Niel, Belgium) [1] for targeted resequencing on a MiSeq sequencing platform (Illumina, San Diego, CA, USA). Using this technology, known pathogenic mutations were excluded in all carriers of compound heterozygous *ATP10B* mutations. Abbreviations: PD, Parkinson's disease; AD, Alzheimer's disease; FTD, frontotemporal dementia; ALS, amyotrophic lateral sclerosis.

Table S6. Control carriers of compound heterozygous *ATP10B* mutant alleles

Control individual	Gender	AAI	ΔCDS[a]	ΔAA[b]	MAF gnomAD (%)	MAF patient cohort (%) n=843	MAF control cohort (%) n=598	F compound heterozygotes in patients (%)	F compound heterozygotes in controls (%)	F compound heterozygotes expected[c] (%)
DR1141	Male	75	c.3086G>A	p.R1029H	0.039	0	0.17	0.0	0.17	0.0015
			c.1177G>T	p.G393W	1.9	1.8	2.5			
DR1508	Female	69	c.2595C>A	p.N865K[#]	2.0	1.8	1.6	0.0	0.17	0.0036
			c.2011G>A	p.G671R[#]	2.0	1.8	1.6			
			c.314A>G	p.N105S	0.30	0.059	0.42			

Note: Control individuals were not assessed for motor symptoms. [a]Coding nomenclature according to NM_025153; [b]Protein nomenclature according to NP_079429; [c]Frequency calculated according to the Hardy–Weinberg principle, using the MAF of single alleles in the PD, DLB and control cohort (n=1441) [#]Variants in *cis* configuration. Abbreviations: ΔCDS, coding sequence substitution; ΔAA, amino acid substitution; MAF, minor allele frequency; F, frequency; AAI, age at inclusion; gnomAD, Genome Aggregation Database [4]

Table S7: Compound heterozygotes with a frequency > 0.01%

Dx	Gender	AAO / AAI	ΔCDS[a]	ΔAA[b]	MAF gnomAD (%)	MAF patient cohort (%) n=843	MAF control cohort (%) n=598	F compound heterozygotes in the corresponding cohort[c] (%)	F compound heterozygotes in the complete cohort[d] (%) n=2047	F compound heterozygotes expected[e] (%)
PD	F	78	c.2595C>A	p.N865K	2.0	1.8	1.6	0.24	0.24	0.037
			c.2011G>A	p.G671R	2.0	1.8	1.6			
			c.1177G>T	p.G393W	1.9	1.8	2.5			
PD	M	81	c.2595C>A	p.N865K	2.0	1.8	1.6	0.24	0.24	0.037
			c.2011G>A	p.G671R	2.0	1.8	1.6			
			c.1177G>T	p.G393W	1.9	1.8	2.5			
MCI	M	61	c.2595C>A	p.N865K	2.0	1.8	1.6	0.34	0.24	0.037
			c.2011G>A	p.G671R	2.0	1.8	1.6			
			c.1177G>T	p.G393W	1.9	1.8	2.5			
Control	M	81	c.2595C>A	p.N865K	2.0	1.8	1.6	0.33	0.24	0.037
			c.2011G>A	p.G671R	2.0	1.8	1.6			
			c.1177G>T	p.G393W	1.9	1.8	2.5			
Control	F	83	c.2595C>A	p.N865K	2.0	1.8	1.6	0.33	0.24	0.037
			c.2011G>A	p.G671R	2.0	1.8	1.6			
			c.1177G>T	p.G393W	1.9	1.8	2.5			
Control	M	62	c.2595C>A	p.N865K	2.0	1.8	1.6	0.17	0.049	N.A.
			c.2011G>A	p.G671R	2.0	1.8	1.6			
			c.1177G>T	p.G393W (hom)	1.9	1.8	2.5			
AD	M	57	c.3665T>C	p.I1222T	1.2	1.3	1.1	0.34	0.098	0.021
			c.2595C>A	p.N865K	2.0	1.8	1.6			
			c.2011G>A	p.G671R	2.0	1.8	1.6			
Control	F	87	c.3665T>C	p.I1222T	1.2	1.3	1.1	0.17	0.098	0.021
			c.2595C>A	p.N865K	2.0	1.8	1.6			
			c.2011G>A	p.G671R	2.0	1.8	1.6			
FTD	M	51	c.3665T>C	p.I1222T	1.2	1.3	1.1	0.31	0.049	0.026
			c.1177G>T	p.G393W	1.9	1.8	2.5			
Control	F	68	c.2595C>A	p.N865K (hom)	2.0	1.8	1.6	0.17	0.049	0.030
			c.2011G>A	p.G671R (hom)	2.0	1.8	1.6			

Note: p.N865Kp.G671R/p.G393W can occur both in *cis* or *trans* configuration. Therefore, the frequency observed in the complete cohort is larger than the frequency expected. The frequency observed for all other combinations is comparable to what is expected according to the Hardy–Weinberg principle. [a]Coding nomenclature according to NM_025153; [b]Protein nomenclature according to NP_079429; [c]PD and DLB cohort (n=643), control cohort (n=598), FTD cohort (n=316) or AD cohort (n=290); [d]Including the PD, DLB, AD, FTD and control cohort (n=2047); [e]Frequency calculated according to the Hardy–Weinberg principle, using the MAF of single alleles in PD, DLB and control cohort, n=1441); Abbreviations: Dx, diagnosis; PD, Parkinson's disease; DLB, dementia with Lewy bodies; MCI, mild cognitive impairment; AD, Alzheimer's disease; FTD, frontotemporal dementia; ΔCDS, coding sequence substitution; ΔAA, amino acid substitution; MAF, minor allele frequency; F, frequency; AAO, age at onset; hom, homozygous; gnomAD, Genome Aggregation Database [4].

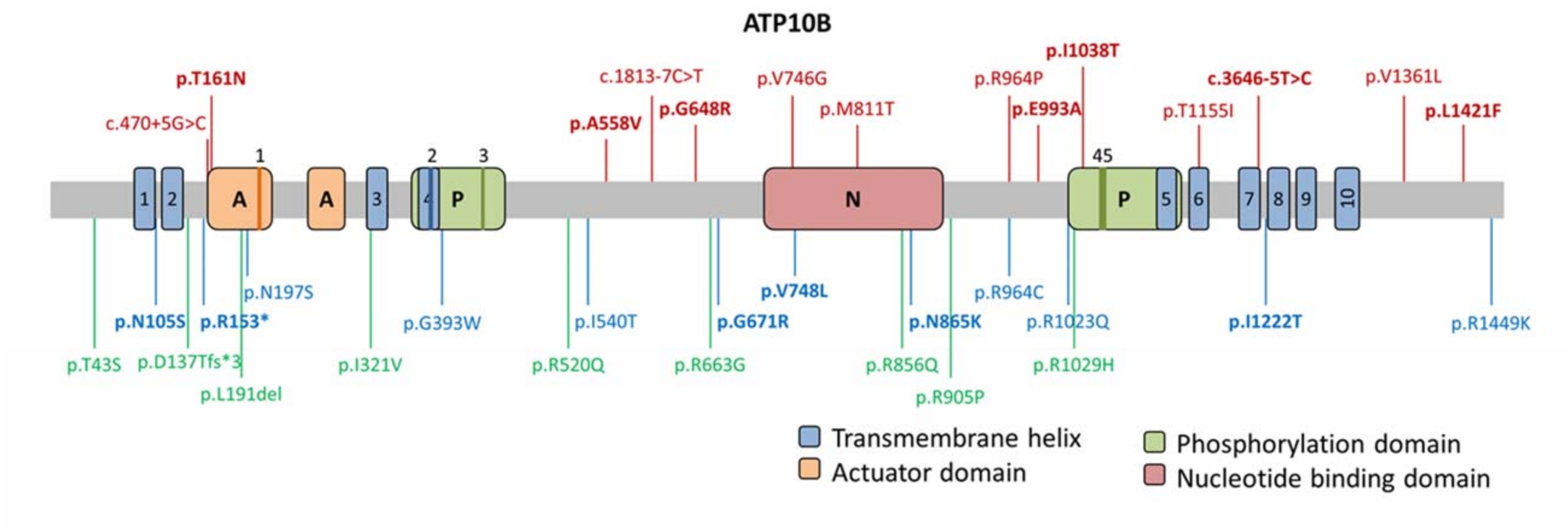

Figure S1: Mutation spectrum of *ATP10B* in PD patients (n=617), DLB patients (n=226) and controls (n=598) cohort

Linear representation of ATP10B indicating all variants identified. Domains are based on data in the InterPro database [3], protein nomenclature according to NP_079429: 1, dephosphorylation site (DGE); 2, substrate binding site (PILS); 3, phosphorylation site (DKT); 4 and 5, Mg^{2+} binding site. Variants in red were observed only in patients, in blue were found in both patients and controls and in green were only present in controls. Variants indicated in bold were found in compound heterozygous patients.

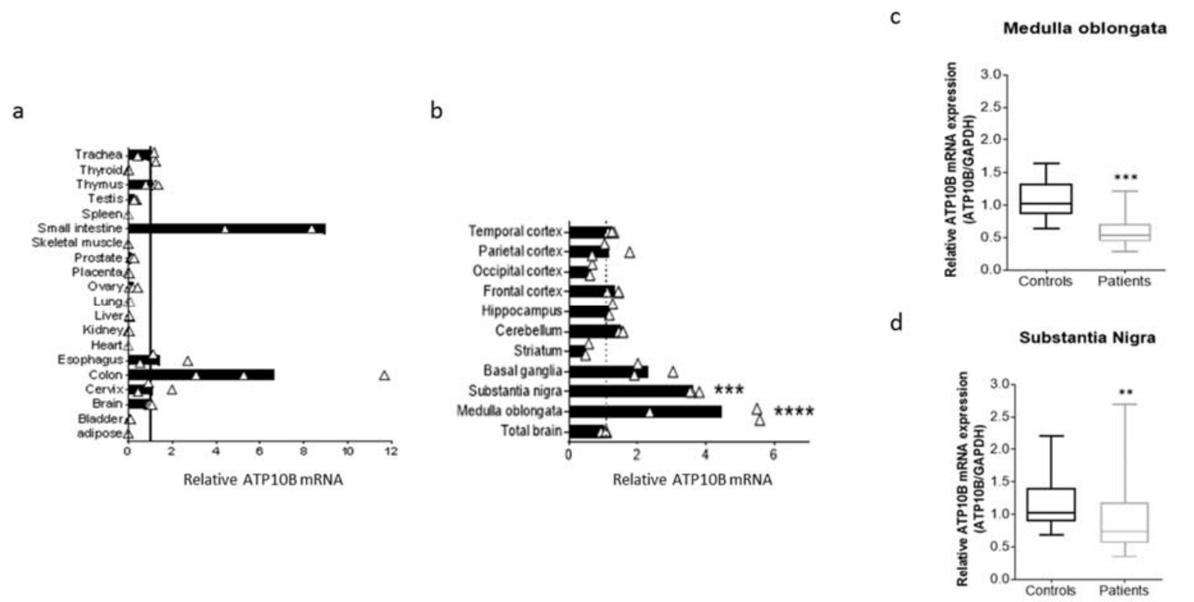

Figure S2. ATP10B mRNA expression in various tissues

a-b. ATP10B mRNA expression was determined by qRT-PCR in commercially available human tissue samples or specific brain regions. These experimental findings are in line with the data in two independent mRNA expression databases [2, 5]. **c-d.** mRNA expression of *ATP10B* in the *medulla oblongata* (c) and *substantia nigra* (d) was assessed by qRT-PCR in available brain samples of control individuals (n=4) and PD patients (n=5). Boxplots depict the relative *ATP10B* mRNA expression levels. Experimental data are the mean of 3 independent experiments and where required ± SEM. For tissue expression analysis (a-b), significance was determined by by One Way ANOVA with Dunnett’s post hoc correction. For c-d, median as well as minimum and maximum values are shown, and statistical significance was calculated using the Mann–Whitney U test. In all cases: ***P< 0.01, ***P<0.001, ****P<0.0001.*

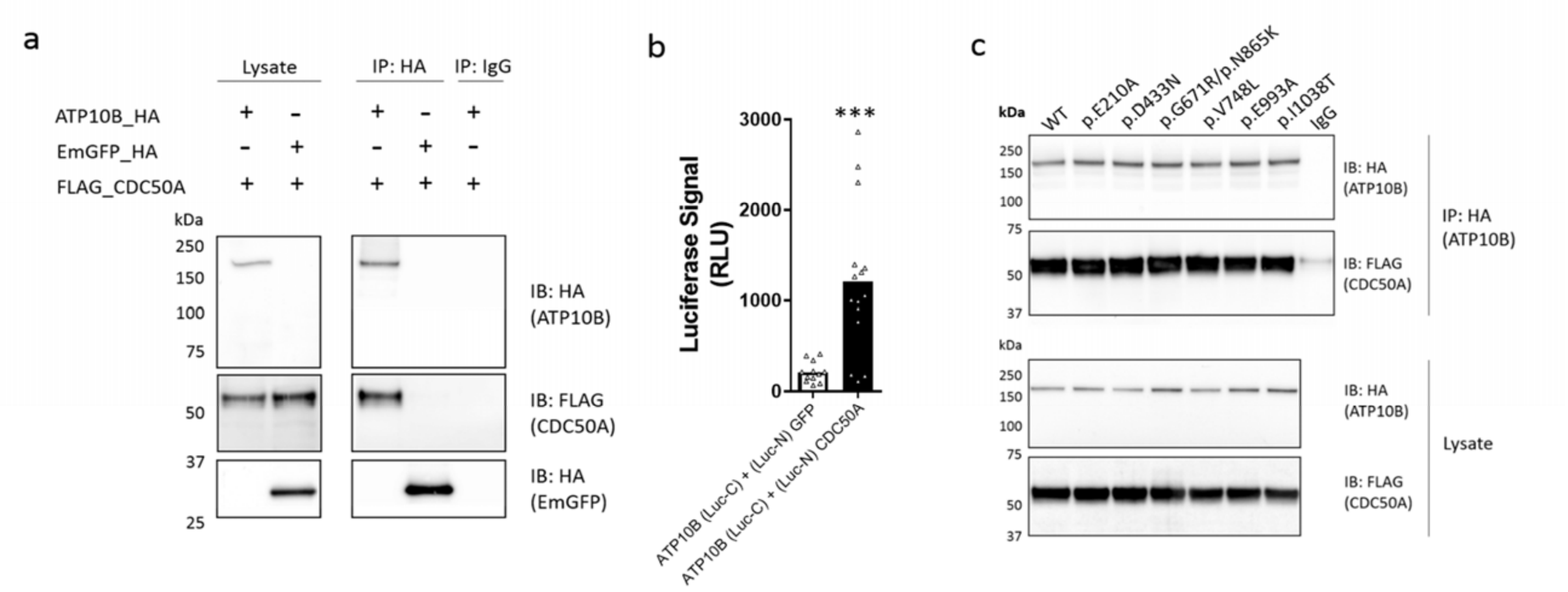

Figure S3. ATP10B interacts with CDC50A

a. Interaction of ATP10B with CDC50A isoform 1 was confirmed by co-immunoprecipitation on HEK293T cells transiently overexpressing both HA-ATP10B WT and 3xFLAG-CDC50A isoform 1. Pull-down was directed against the HA-tag of ATP10B and the presence of CDC50A isoform 1 was analyzed by immunoblotting with an antibody against the 3xFLAG tag. Both EmGFP-HA and FLAG beads (IgG only) were used as a negative control. **b.** Protein complementation assay using split luciferase constructs confirms the interaction between ATP10B and CDC50A. HEK293T cells were transiently co-transfected with split luciferase fusion constructs of ATP10B, CDC50A isoform 1 and GFP as a control. Luc-N and Luc-C are respectively the N- and C-terminal part of luciferase. The position of Luc-N/Luc-C in the construct name (front or back) corresponds to the fusion position of the luciferase fragment in the construct (N- or C-terminal). **c**. The interaction between CDC50A isoform 1 and ATP10B was also confirmed for the catalytic and disease-associated variants via co-immunoprecipitation as in panel (a). HEK293T cells were transiently co-

transfected with FLAG tagged CDC50A isoform 1 and HA-tagged ATP10B WT, catalytic mutants p.E210A and p.D433N, as well as the identified variants p.G671R/p.N865K, p.V748L, p.E993A and p.I1038T. IgG only was used as a negative control. Immunoblots are representative examples of 3 experiments and bar graph depicts the average relative luminescent units (RLU) ± SEM of 3 independent experiments. Significance was calculated using an unpaired t test analysis, ***P<0.001.

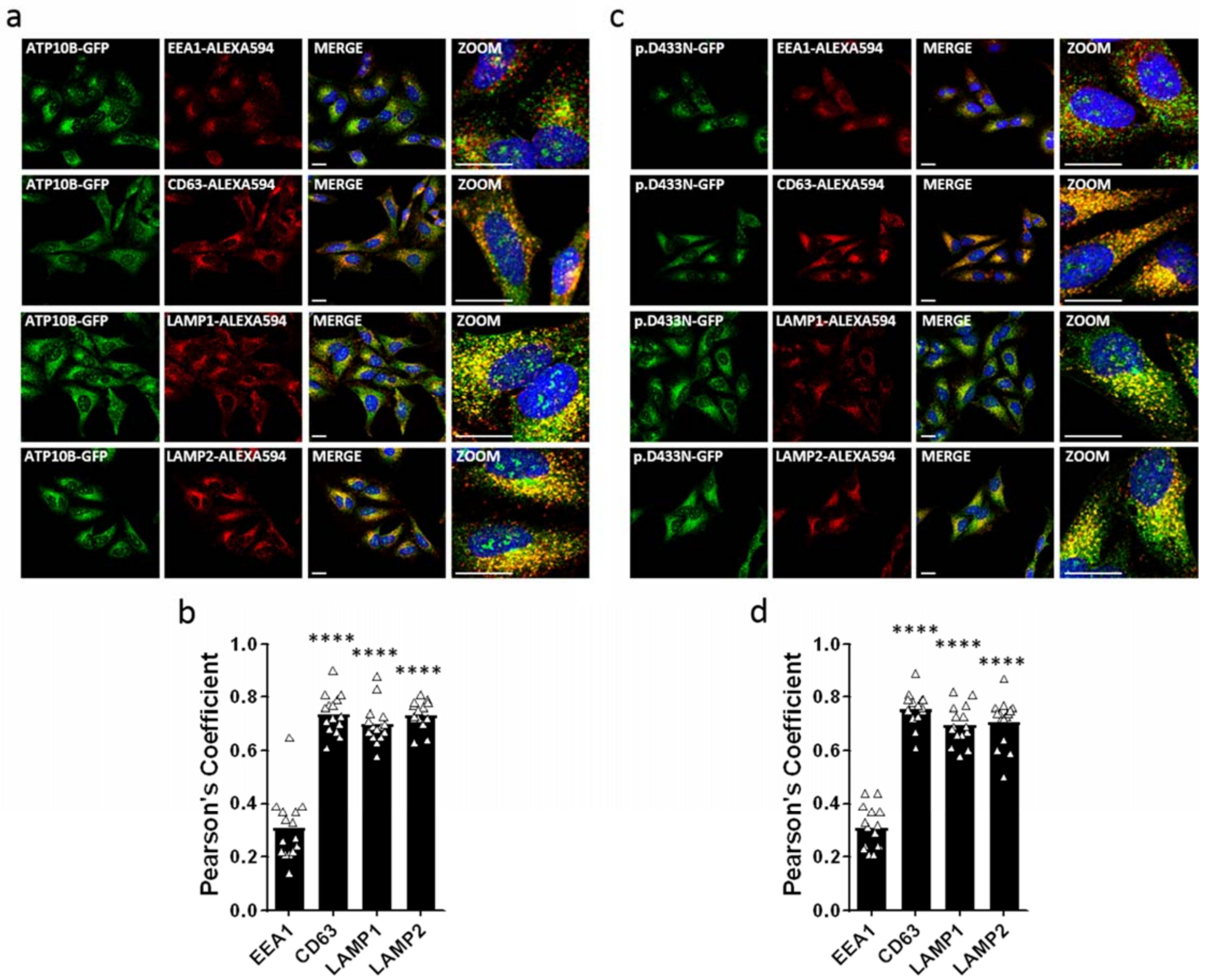
a
ATP10B-GFP
EEA1-ALEXA594
MERGE
ZOOM
ATP10B-GFP
CD63-ALEXA594
MERGE
ZOOM
ATP10B-GFP
LAMP1-ALEXA594
MERGE
ZOOM
ATP10B-GFP
LAMP2-ALEXA594
MERGE
ZOOM
c
p.D433N-GFP
EEA1-ALEXA594
MERGE
ZOOM
p.D433N-GFP
CD63-ALEXA594
MERGE
ZOOM
p.D433N-GFP
LAMP1-ALEXA594
MERGE
ZOOM
p.D433N-GFP
LAMP2-ALEXA594
MERGE
ZOOM
b
Pearson's Coefficient
1.0
0.8
0.6
0.4
0.2
0.0
****
****
****
EEA1
CD63
LAMP1
LAMP2
d
Pearson's Coefficient
1.0
0.8
0.6
0.4
0.2
0.0
****
****
****
EEA1
CD63
LAMP1
LAMP2

Figure S4. ATP10B WT and the p.D433N mutant localize to the late endo-/lysosomes

a-d. The subcellular localization of ATP10B was determined in HeLa cells stably overexpressing CDC50A in combination with ATP10B WT-GFP (a-b) or p.D433N-GFP (c-d). Cells were stained with intracellular markers (EEA1, early endosomes; CD63, late endosomes; LAMP1, late endo-/lysosomes; LAMP2, lysosomes) to assess co-localization via confocal microscopy. Scale bar = 10 µm. Images are representative of 3 independent experiments. Bar graphs depict the average Pearson's coefficient ± SEM. Differences relative to the EEA1 Pearson's coefficients were assessed by One Way ANOVA's with Dunnett's post hoc correction to EEA1 whereby; **** P<0.0001.

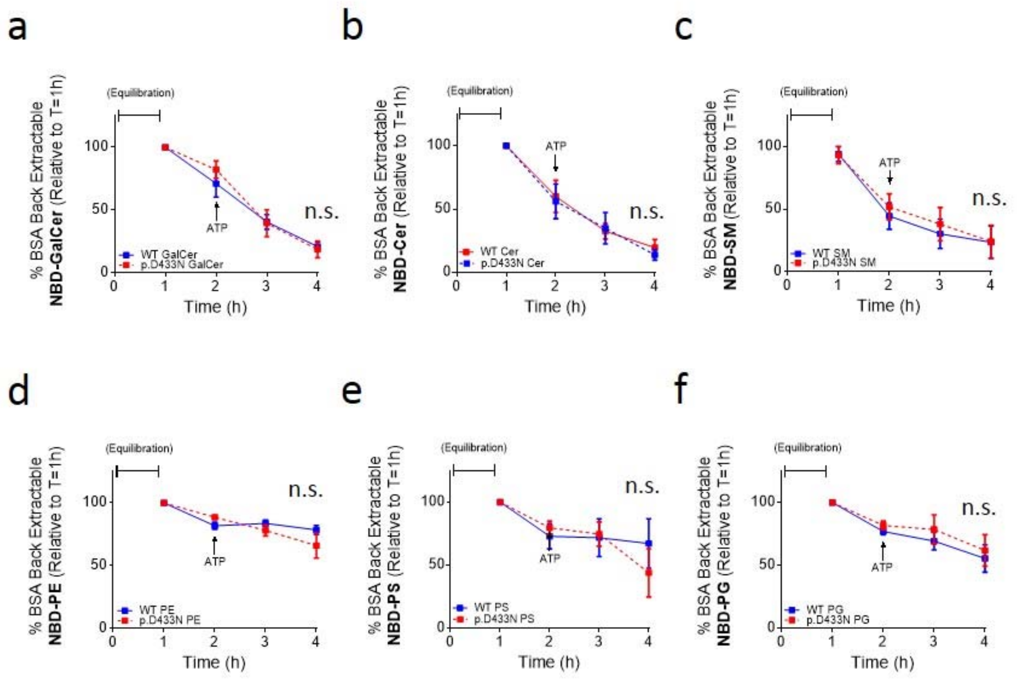

Figure S5. ATP10B lipid specificity

a-f. Microsomes of HeLa cells with stable overexpression of CDC50A alone or in combination with ATP10B WT or the catalytic p.D433N mutant were assessed for ATP10B expression and activity. Cells overexpressing CDC50A alone or co-expressing the catalytic mutant p.D433N were used as negative controls. Microsomes were assessed for their potential to translocate nitrobenzoxadiazole (NBD)-labeled phosphatidylethanolamine (PE, a), phosphatidylserine (PS, b), phosphatidylglycerol (PG, c), galactosylceramide (GalCer, d), ceramide (Cer, e) or sphingomyelin (SM, f) over time (0-4 h). We followed the lipid translocation by determining the fraction of fluorescent NBD-labeled lipids that is extractable from the accessible cytosolic membrane leaflet by fatty acid free bovine serum albumin. First, we observed spontaneous translocation of fluorescent labeled lipids from the cytosolic to exoplasmic membrane leaflet (1-2 h). Following 1 mM ATP addition (at the 2 h time point, *i.e.* 2 h after the membranes were

placed at 37°C), lipid translocation was followed from the extra-cytosolic to cytosolic membrane leaflet. For the lipid translocation assay, differences were assessed by Two Way ANOVA whereby; n.s. = non-significant.

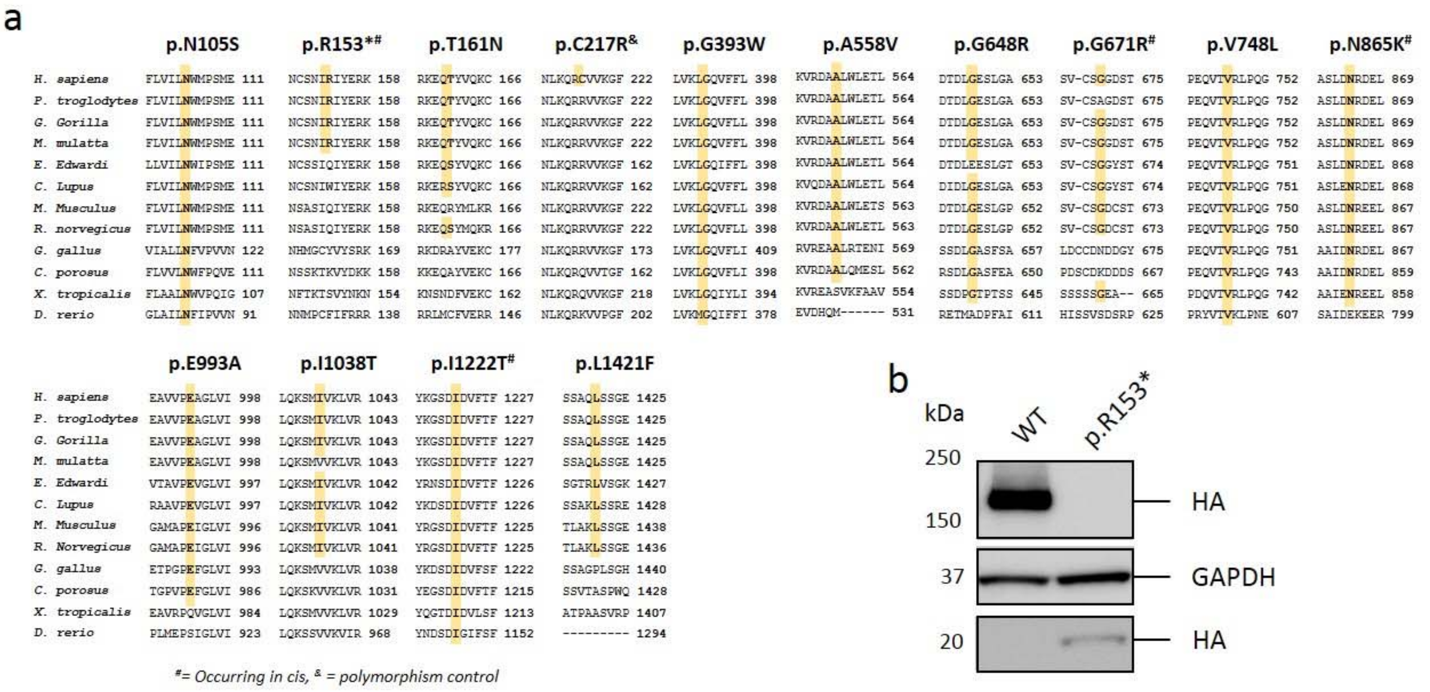

Figure S6. Expression levels and ATPase activity of ATP10B variants in stable cell lines

a. Sequence alignments and residue conservation of the ATP10B protein variants p.N105S, p.R153*, p.T161N, p.G393W, p.A558V, p.G648R, p.G671R, p.N865K, p.V748L, p.E993A, p.I1038T, p.I1222T, p.L1421F and &the polymorphism p.C217R; #are on the same haplotype, *in cis* configuration (sequence NM_025153). Yellow depicts conservation across species. **b.** Due to the unavailability of an antibody targeting the N-terminus of ATP10B, we confirmed the capacity to express ATP10B p.R153* mutant via transient transfection of ATP10B WT and p.R153* HA-fusion constructs in HEK293T cells. Following transfection, samples were subjected to immunoblot analysis for HA, using GAPDH as a loading control. p.R153* is detected significantly lower (HA lower panel, lower molecular weight) than ATP10B WT (HA upper panel), most likely due to a reduced protein stability as a consequence of the truncation.

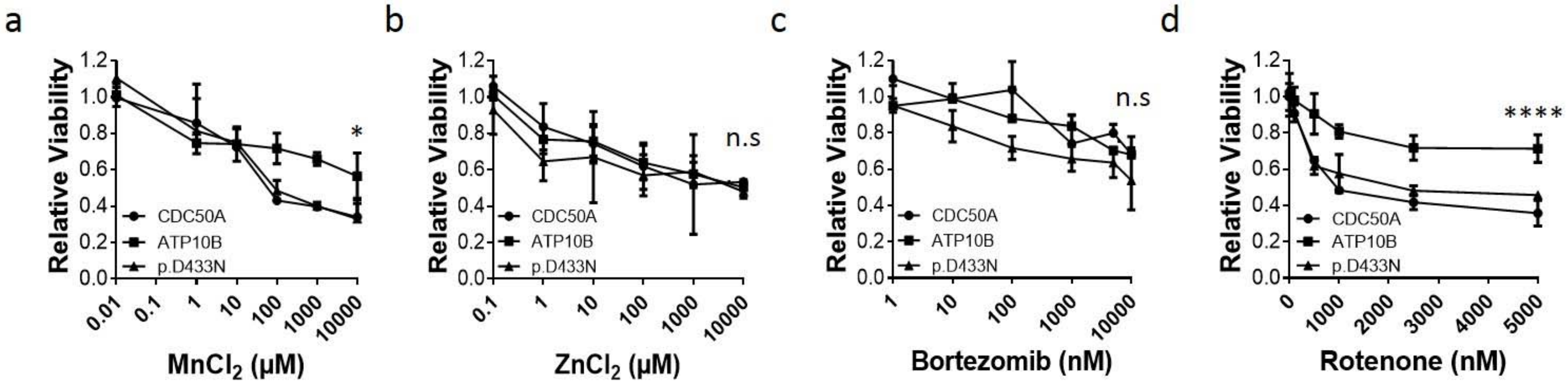

Figure S7. ATP10B provides cellular protection to PD-related stressors

a-d HeLa cells stably overexpressing CDC50A alone or combined with ATP10B WT or p.D433N were exposed for 48 h to increasing doses of various PD-related stressors ($MnCl_2$, 0-10 mM, a.; $ZnCl_2$, 0-10 mM, b.; Bortezomib, 0-10 µM, c.; or rotenone, 0-5 µM, d). Cell toxicity was assessed by a MUH (4-methylumbelliferyl heptanoate) viability assay. Data are the average ± SEM of 3 independent experiments and are expressed relative to the control (vehicle treatment). Significance of panels (a-d) was assessed by Two Way ANOVA whereby, *P<0.05 and ****P<0.0001. n.s. = not significant.

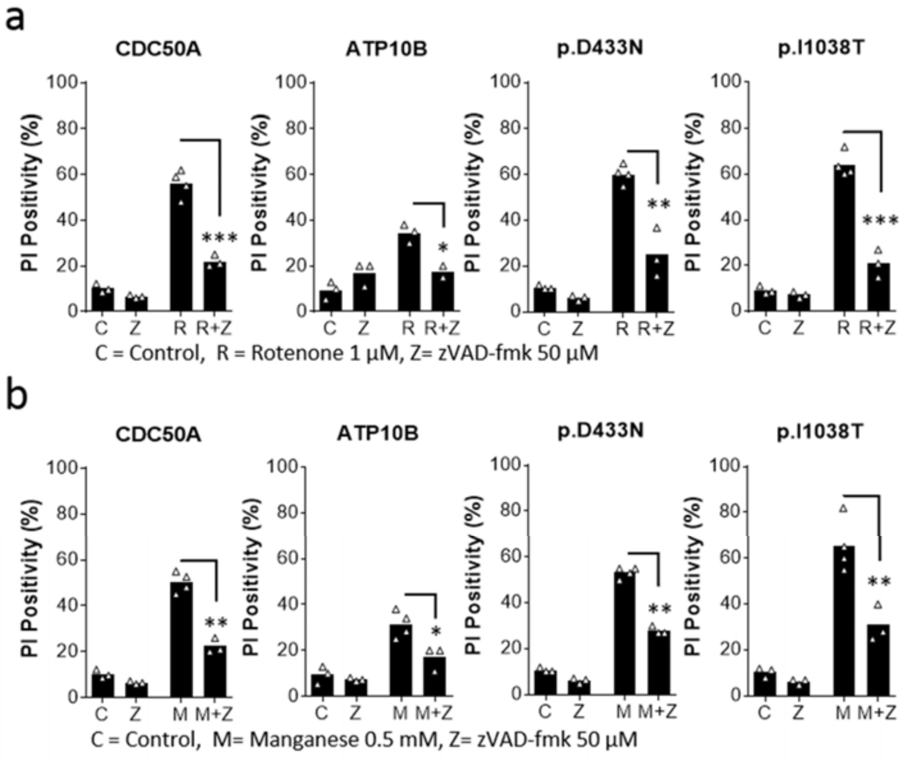

Figure S8. ATP10B protects against stress induced apoptosis

a-b. The caspase inhibitor Zvad-fmk (Z, 50 nM; 1 h pretreatment; 37°C) reduces the cell death observed in the stable HeLa cell models expressing ATP10B variants during rotenone (R) or manganese (M) exposure, indicating that cell toxicity is explained by the induction of apoptosis. Cell toxicity was assessed by propidium iodide (PI) exclusion (1 µg/ml, 5 min) based flow cytometry. Data are the average ± SEM of 3 independent experiments. Significance of Zvad-fmk was assessed by One Way ANOVA with Dunnett's post hoc to R. *P<0.05, **P<0.01, ***P<0.001.

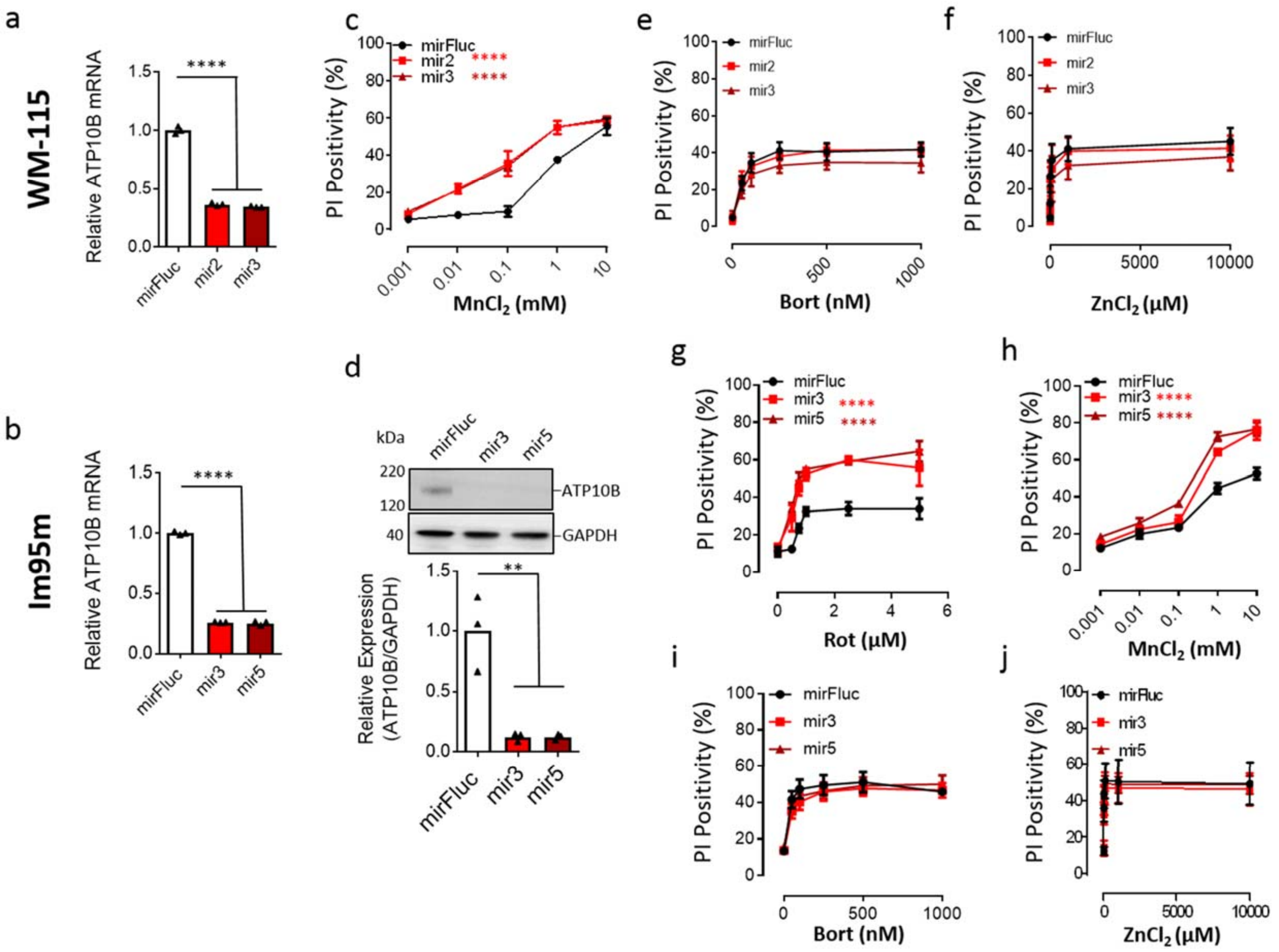

a
b
c
d
e
f
g
h
i
j
WM-115
lm95m
Relative ATP10B mRNA
mirFluc
mir2
mir3
mir5
****
**
PI Positivity (%)
MnCl2 (mM)
Bort (nM)
ZnCl2 (µM)
Rot (µM)
kDa
220
120
40
ATP10B
GAPDH
Relative Expression (ATP10B/GAPDH)

Figure S9. ATP10B knockdown sensitizes cell models to rotenone and manganese toxicity

Two human cell lines with endogenous ATP10B expression were identified, the melanoma WM-115 (a) and adenocarcinoma Im95m (b) cells. ATP10B expression was not detected in commonly used neuroblastoma cell models. WM-115 and Im95m cells were subjected to ATP10B knockdown. Two independent microRNA based short-hairpins (mir) were used to generate stable knockdown cells. mirFluc serves as a control cell line that was transduced with a mir against the firefly luciferase gene (Fluc). qRT-PCR analysis confirms expression and knockdown of ATP10B mRNA in WM-115 (a) and Im95m cells (b) in relation to β-actin as a reference gene. Knockdown at the ATP10B protein level was also confirmed in both the Im95m (d) and WM115 cells (Fig. 3a-b) by immunoblot analysis for ATP10B in comparison to GAPDH as loading control. A >80% decreased ATP10B protein expression was found in the Im95m and WM115 knockdown cell lines. The effect of ATP10B knockdown was assessed on the sensitivity of WM-115 and Im95m cells to increasing concentrations of rotenone (Rot, Fig. 4c and g), manganese ($MnCl_2$, c and h), Bortezomib (Bort, e and i) or zinc ($ZnCl_2$, f and j). Propidium iodide (PI) exclusion (1 µg/ml, 5 min) based flow cytometry was used as a readout for cell death. Statistical validation of ATP10B knockdown was assessed by One Way ANOVA with Dunnett's post hoc to mirFluc and differences between cell line sensitivities were assessed by Two Way ANOVA's whereby; * $P<0.05$, **$P<0.01$, ****$P<0.0001$.

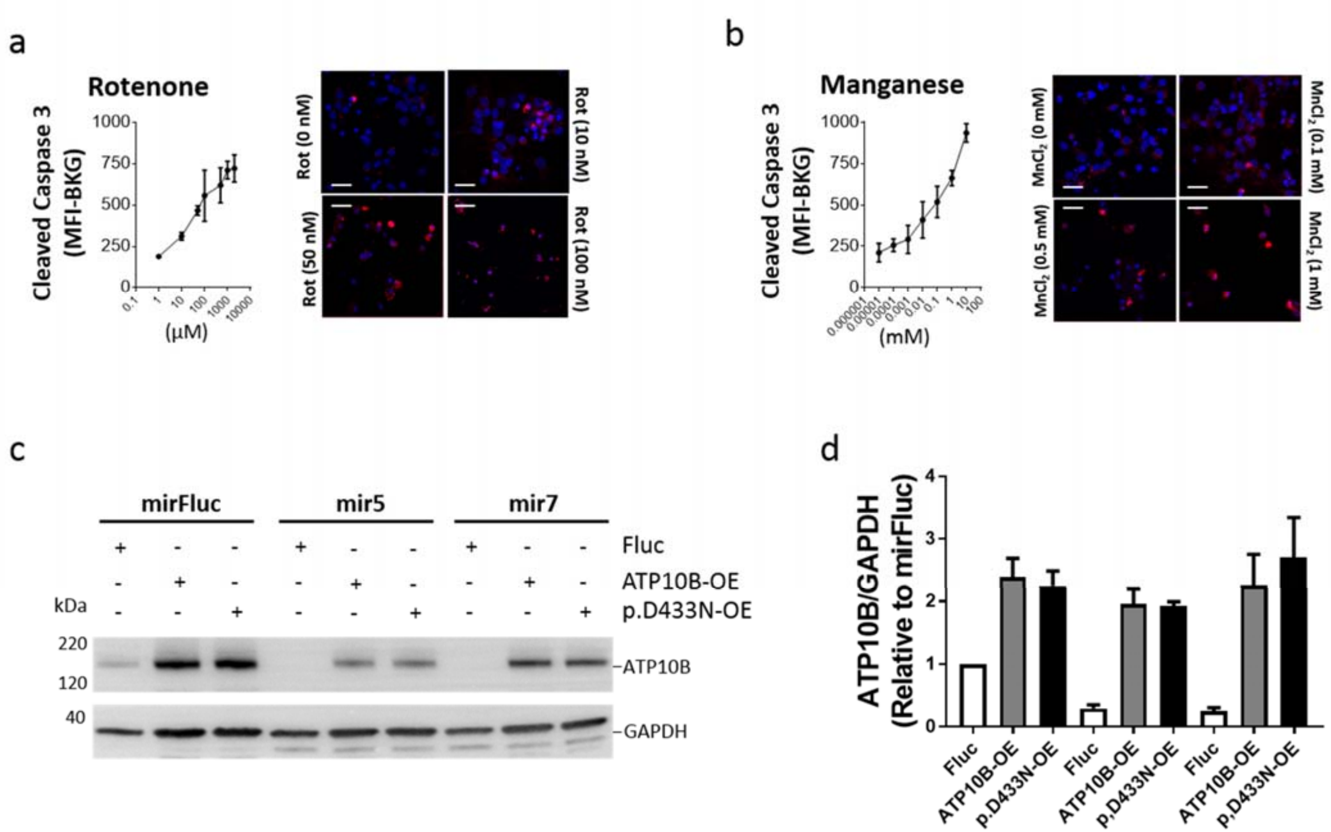

Figure S10. Cell stress optimization and ATP10B knockdown and recovery in cortical neurons

a-b. Cortical neurons were exposed to increasing doses of rotenone (Rot, 0-2 µM, a) and manganese ($MnCl_2$, 0-10 mM, b) for 48 h and induction of apoptosis was assayed via flow cytometry and confirmed by confocal microscopy using an antibody directed against cleaved caspase 3. **c.** Immunoblot analysis and subsequent densitometry of isolated cortical neurons transduced with ATP10B targeting mir's (mir5 and mir 7) in comparison to control and following reintroduction of human ATP10B or pD433N variants in comparison to Fluc. GAPDH was used as a loading control.

REFERENCES

1. Goossens D, Moens LN, Nelis E, Lenaerts AS, Glassee W, Kalbe A et al. (2009) Simultaneous mutation and copy number variation (CNV) detection by multiplex PCR-based GS-FLX sequencing. Hum Mutat 30:472-476. doi:10.1002/humu.20873
2. Hruz T, Laule O, Szabo G, Wessendorp F, Bleuler S, Oertle L et al. (2008) Genevestigator v3: a reference expression database for the meta-analysis of transcriptomes. Adv Bioinformatics 2008:420747. doi:10.1155/2008/420747
3. Hunter S, Apweiler R, Attwood TK, Bairoch A, Bateman A, Binns D et al. (2009) InterPro: the integrative protein signature database. Nucleic Acids Res 37:D211-215. doi:10.1093/nar/gkn785
4. Lek M, Karczewski KJ, Minikel EV, Samocha KE, Banks E, Fennell T et al. (2016) Analysis of protein-coding genetic variation in 60,706 humans. Nature 536:285-291. doi:10.1038/nature19057
5. Uhlen M, Fagerberg L, Hallstrom BM, Lindskog C, Oksvold P, Mardinoglu A et al. (2015) Proteomics. Tissue-based map of the human proteome. Science 347:1260419. doi:10.1126/science.1260419
